# Butyrophilin-like 3 Directly Binds a Human Vγ4^+^ T Cell Receptor Using a Modality Distinct from Clonally-Restricted Antigen

**DOI:** 10.1016/j.immuni.2019.09.006

**Published:** 2019-11-19

**Authors:** Carrie R. Willcox, Pierre Vantourout, Mahboob Salim, Iva Zlatareva, Daisy Melandri, Leonor Zanardo, Roger George, Svend Kjaer, Mark Jeeves, Fiyaz Mohammed, Adrian C. Hayday, Benjamin E. Willcox

**Affiliations:** 1 Institute of Immunology and Immunotherapy, University of Birmingham, Birmingham, UK; 2 Cancer Immunology and Immunotherapy Centre, University of Birmingham, Birmingham, UK; 3 Peter Gorer Department of Immunobiology, School of Immunology and Microbial Sciences, King’s College London, London, UK; 4 Immunosurveillance Laboratory, The Francis Crick Institute, London, UK; 5 Faculty of Medicine, University of Tours, Tours, France; 6 Structural Biology Team, The Francis Crick Institute, London, UK; 7 Henry Wellcome Building for NMR, Institute of Cancer and Genomic Sciences, University of Birmingham, Birmingham, UK

**Keywords:** butyrophilin, gamma delta T cell, T cell receptor, complementarity determining region, ligand, selection

## Abstract

Butyrophilin (BTN) and butyrophilin-like (BTNL/Btnl) heteromers are major regulators of human and mouse γδ T cell subsets, but considerable contention surrounds whether they represent direct γδ T cell receptor (TCR) ligands. We demonstrate that the BTNL3 IgV domain binds directly and specifically to a human Vγ4^+^ TCR, “LES” with an affinity (∼15–25 μM) comparable to many αβ TCR-peptide major histocompatibility complex interactions. Mutations in germline-encoded Vγ4 CDR2 and HV4 loops, but not in somatically recombined CDR3 loops, drastically diminished binding and T cell responsiveness to BTNL3-BTNL8-expressing cells. Conversely, CDR3γ and CDR3δ loops mediated LES TCR binding to endothelial protein C receptor, a clonally restricted autoantigen, with minimal CDR1, CDR2, or HV4 contributions. Thus, the γδ TCR can employ two discrete binding modalities: a non-clonotypic, superantigen-like interaction mediating subset-specific regulation by BTNL/BTN molecules and CDR3-dependent, antibody-like interactions mediating adaptive γδ T cell biology. How these findings might broadly apply to γδ T cell regulation is also examined.

## Highlights

• BTNL3 binds directly and specifically to Vγ4^+^ TCRs via its IgV domain
• The superantigen-like binding mode focuses on germline-encoded TCR regions
• In contrast, γδ TCR binding to a clonally restricted antigen is CDR3-mediated
• Mutagenesis indicates parallels with BTN3A1-mediated activation of Vγ9Vδ2 T cells

## Graphical Abstract

**Figure:**
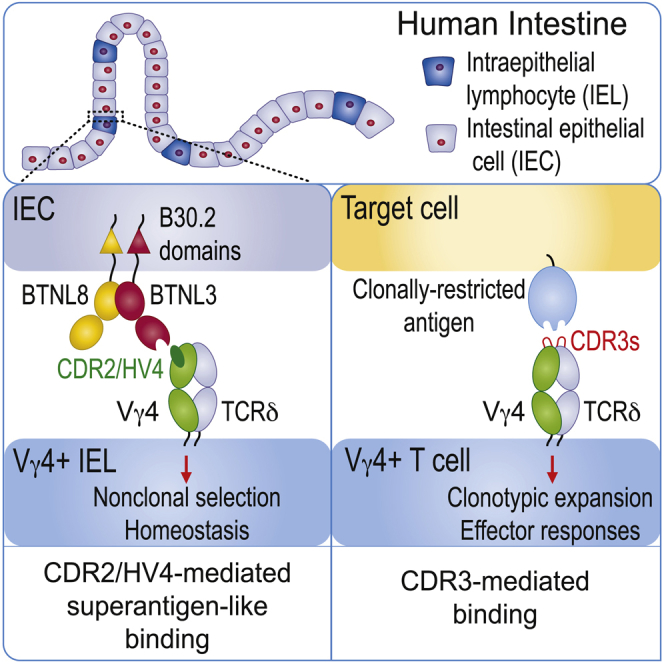

## Teaser

Butyrophilin (BTN) and butyrophilin-like (BTNL) molecules powerfully influence selection and activation of specific γδ lymphocyte subsets, but whether they directly bind the γδ TCR has remained contentious. Willcox et al. show that BTNL3 directly binds to human Vγ4^+^ TCRs via a superantigen-like binding mode that is focused on germline-encoded TCR regions.

## Introduction

γδ T cells seemingly make both innate-like and adaptive contributions to immunity, with increasingly appreciated relevance to clinical scenarios such as cancer surveillance. Prototypic innate-like γδ T cell subtypes include mouse dendritic epidermal T cells (DETCs), which are skin-restricted, feature a canonical T cell receptor (TCR) repertoire (Asarnow et al., 1988), and mediate responses to dysregulated target cells in the absence of foreign adjuvants or antigens (Strid et al., 2008). Indeed, DETC-deficient mice show increased susceptibility to skin carcinogenesis (Girardi et al., 2001). In humans, a limited TCR repertoire is likewise expressed by a major subset of Vγ9Vδ2 T cells (Delfau et al., 1992), which are preferentially enriched in peripheral blood, display an effector phenotype (Parker et al., 1990), and show potent cytotoxicity and cytokine production. Given that they respond *en masse* to microbial phosphoantigens (P-Ags) (Morita et al., 2007), the Vγ9Vδ2 subset likely provides an early line of defense against certain microbial infections, such as those involving eubacterial and mycobacterial species that produce the highly potent P-Ag (E)-4-hydroxy-3-methyl-but-2-enyl pyrophosphate (HMBPP). Conversely, adaptive paradigms seem most able to explain conspicuous clonal expansions and effector differentiation of subsets of human Vδ2^neg^ T cells and Vγ9^neg^Vδ2 T cells, including after exposure to viral infection (Davey et al., 2017, Davey et al., 2018b, Ravens et al., 2017).

Few direct ligands of the γδ TCRs underpinning innate-like or adaptive responses are known. Adaptive processes highlight powerful clonotypic focusing even within specific V region subsets (e.g., Vδ1 T cells, Vδ1^neg^Vδ2^neg^ T cells, and Vγ9^neg^Vδ2 T cells), strongly suggesting that somatically recombined CDR3 regions are involved (Davey et al., 2018a). Moreover, a diverse range of ligands has been proposed for such populations, including those few supported by evidence of direct TCR-ligand interaction, many of which favor roles for CDR3 residues (Willcox and Willcox, 2019).

At the same time, molecules closely related to the B7 family of lymphocyte co-regulators (which include CD80, ICOS-L, and PDL1) have emerged as critical players in γδ T cell selection, activation, and possibly tissue-associated functions (Abeler-Dörner et al., 2012). The first of these to be identified was Skint1, a hitherto uncharacterized BTNL molecule crucial for thymic selection of Vγ5^+^ DETC and expressed by keratinocytes (Boyden et al., 2008). Subsequently, expression of the human BTN3A1 molecule on target cells was established as critical for P-Ag-mediated activation of human peripheral blood Vγ9Vδ2^+^ T cells (Harly et al., 2012, Vavassori et al., 2013). More recently, mouse Btnl1 emerged as critical for the extrathymic selection of the signature Vγ7^+^ intestinal intraepithelial lymphocyte (IEL) population (Di Marco Barros et al., 2016). Btnl1 and Btnl6 molecules are both expressed by differentiated enterocytes (Di Marco Barros et al., 2016), wherein they form a co-complex (Lebrero-Fernández et al., 2016a, Vantourout et al., 2018) that can specifically regulate mature Vγ7^+^ IEL *in vitro*. Likewise, human BTNL3 and BTNL8, which are both enriched in expression in gut epithelium (Di Marco Barros et al., 2016, Lebrero-Fernández et al., 2016b), are major regulators of signature human intestinal Vγ4^+^ T cells (Di Marco Barros et al., 2016, Melandri et al., 2018) and are capable of inducing TCR downregulation specifically in this subset. The potential pathophysiologic significance of the Vγ4^+^ T cell subset was highlighted recently by a report that disruption of the Vγ4^+^-BTNL3.8 axis is pathognomonic for celiac disease (Mayassi et al., 2019). More generally, TCR γδ IELs have been increasingly implicated in the regulation of tissue maintenance, including protection from infection, inflammation, and internal dysregulation (Edelblum et al., 2015, He et al., 2019, Hoytema van Konijnenburg et al., 2017). Thus, deficiencies in signature, tissue-resident γδ T cell compartments have been causally linked to cancer and tissue inflammation (Girardi et al., 2002, Roberts et al., 1996, Strid et al., 2008).

Soon after their discovery, when γδ T cells were first found to express a limited number of V regions, the existence of a range of host-encoded ligands that might mediate subset-specific γδ T cell selection and/or activation was hypothesized (Janeway et al., 1988). Clearly, the observations outlined earlier highlight BTN and BTNL molecules as strong candidates for being direct, subset-specific γδ TCR ligands. Nevertheless, the only study reporting direct binding of a TCR (Vγ9Vδ2) to a BTN or BTNL molecule (BTN3A1) (Vavassori et al., 2013) has been strongly disputed (Sandstrom et al., 2014), with its claim of P-Ag presentation by the BTN3A1 V domain being ascribed to electron density arising from crystallization components (Sandstrom et al., 2014). Indeed, other data demonstrate that BTN3A1 can directly bind P-Ag in its C-terminal B30.2 domain (Hsiao et al., 2014, Salim et al., 2017, Sandstrom et al., 2014). Altogether, compelling evidence that any BTN, BTNL, or Btnl molecule acts as a direct ligand for the γδ TCR is lacking, leaving uncertainty about how these molecules may achieve their profound biological effects. Indeed, the possibility has remained that BTN and BTNL molecules may act indirectly, for example, as chaperones or inducers for direct TCR ligands that are as yet unidentified.

Here we provide unequivocal evidence for direct binding of a γδ TCR to a BTNL protein. We show that a human Vγ4 TCR binds the BTNL3 IgV domain via germline-encoded regions, somewhat analogous to superantigen binding to an αβ TCR. In contrast, binding of a clonally restricted ligand to the same Vγ4 TCR was critically influenced by the CDR3 regions of the γδ TCR, consistent with its adaptive biology. Thus, we highlight two distinct and complementary modalities for ligand interaction: one involving a BTNL molecule in Vγ region-specific regulation of tissue-restricted γδ T cell subsets and the other involving highly specific, clonally restricted ligand recognition, underpinning adaptive γδ T cell responses. Moreover, the two binding modes may extend to BTN3A1-mediated regulation of the human blood Vγ9Vδ2 subset by P-Ags, suggesting broad significance of the BTNL modality that we outline.

## Results

### The BTNL3 IgV Domain Directly and Specifically Binds Vγ4 TCRs

Previously, we demonstrated that exposure of Jurkat cells transduced with Vγ4^+^ γδ TCRs to 293T cells expressing BTNL3.8 heterodimers (293T.L3L8) led to CD69 upregulation and TCR downregulation, consistent with TCR triggering (Melandri et al., 2018). Moreover, soluble Vγ4 TCRs were found to specifically stain the surface of 293T.L3L8 target cells, but not control cells transduced with empty vector (293T.EV), suggesting that BTNL3.8 heterodimers either were Vγ4 TCR ligands or induced the display of as-yet-unidentified Vγ4 TCR ligands. Consistent with either possibility, mutagenesis of the BTNL3.8 heterodimer showed that Vγ4-mediated TCR triggering depended on the BTNL3 IgV domain.

To address the hypothesis that BTNL heterodimers directly bound the TCR, we generated recombinant BTNL3 and BTNL8 IgV domains and tested interaction with a range of soluble γδ TCRs (Willcox et al., 2012) using surface plasmon resonance (SPR). We overexpressed both BTNL IgV domains separately in *E. coli* and then renatured them by dilution refolding, with yields broadly similar to those of other B7-like IgV domains, such as Skint1 (Salim et al., 2016) (STAR Methods). Of note, BTNL3 IgV was highly susceptible to oxidation when solubilized in denaturant, and its correct refolding depended on full reduction before refolding and choice of oxido-reduction couple during renaturation. Refolding was also impaired by some C-terminal tag sequences, although not by a 6×His tag.

**Figure 1:**
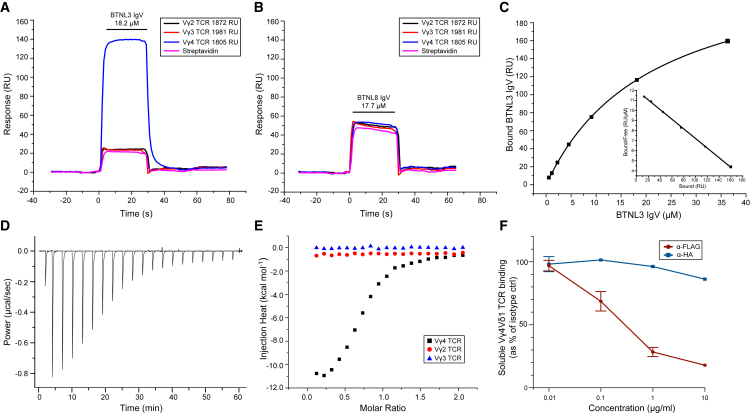
Human BTNL3 IgV Binds Specifically to Vγ4 TCRs (A and B) SPR analysis of BTNL3 IgV (A; 18.2 μM) or BTNL8 IgV (B; 17.7 μM) injected (small horizontal bar) over biotinylated Vγ4 TCR (1,805 RU), Vγ3 TCR (1,981 RU), or Vγ2 TCR (1,872 RU) or streptavidin alone. Responses presented as resonance units (RUs). Data are representative of 15 experiments (A) or two experiments (B). (C) Equilibrium affinity analysis of the binding of BTNL3 IgV to Vγ4 TCR (K_d_ = 22.1 μM); inset, Scatchard plot of the same data (K_d_ = 20.9 μM). (D) ITC analysis of the BTNL3 IgV domain interaction with Vγ4 TCR (K_d_ = 3.5 μM). (E) ITC analysis indicates no interaction of the BTNL3 IgV domain with control Vγ2^+^ or Vγ3^+^ TCRs. (F) Quantitation of effects of anti-FLAG and anti-HA antibodies on the staining of 293T cells expressing FLAG-BTNL3 and HA-BTNL8 with soluble Vγ4^+^ TCR and anti-His monoclonal antibody (mAb). Data are from three independent experiments (mean ± SD). See also Figure S1.

Injection of BTNL3 over immobilized Vγ4 TCR resulted in substantially greater signals than over immobilized Vγ2 or Vγ3 TCRs, indicating Vγ4-specific TCR binding (Figure 1A). In contrast, signals resulting from injection of BTNL8 IgV over surfaces with immobilized Vγ2, Vγ3, and Vγ4 TCRs matched those over control surfaces, indicating that in contrast to BTNL3 IgV, BTNL8 IgV did not directly bind the Vγ4 TCR (Figure 1B). This was consistent with genetic data implicating BTNL3 more than BTNL8 in promoting Vγ4 TCR triggering (Melandri et al., 2018). Equilibrium binding measurements (Figure S1A) indicated the affinity (K_d_) of BTNL3 IgV for a Vγ4 TCR, LES, was ∼15–25 μM (average 20.7 ± 4.8 μM, n = 15) at 25°C (Figure 1C; Figure S1A). Isothermal titration calorimetry (ITC) measurements confirmed Vγ4 TCR specifically bound to BTNL3 IgV, with a broadly similar affinity (3.5 μM at 20°C), and indicated the interaction was enthalpically driven (ΔH° = −8.1 kcal.mol^−1^ at 20°C) and marginally entropically unfavorable (TΔS° = −0.77 kcal.mol^−1^ at 20°C) (Figures 1D and 1E). In contrast, no binding was observed with a Vγ2^+^ or Vγ3^+^ TCR (Figure 1E; Figures S1B and S1C).

Consistent with our finding that BTNL3 IgV, but not BTNL8 IgV, directly bound Vγ4 TCRs, soluble Vγ4 TCR binding to 293T cells transduced with FLAG (N-terminal)-BTNL3 and hemagglutinin (HA) (N-terminal)-BTNL8 heterodimer constructs was abrogated by anti-FLAG antibody, presumably because of steric hindrance, but was only marginally affected by anti-HA antibody (Figure 1F; Figure S1D). Anti-FLAG antibody, but not anti-HA antibody, also inhibited activation of JRT3 Vγ4Vδ1 TCR transductants by 293T cells transduced with FLAG-BTNL3.HA-BTNL8 (Figures S1E–S1G).

**Figure 2:**
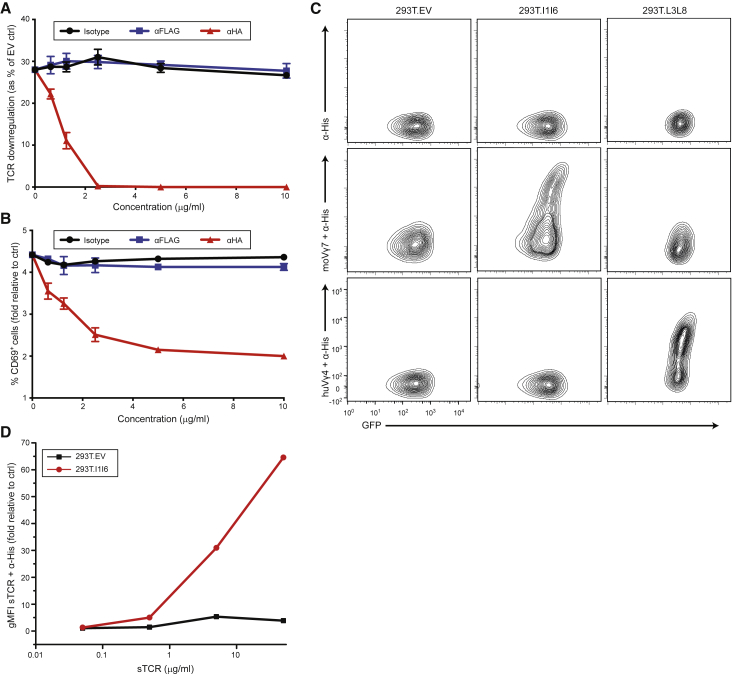
Mouse Vγ7 TCR-Dependent Recognition of Btnl1.6 (A and B) Flow cytometry analysis of TCR downregulation (A) and CD69 upregulation (B) by Jurkat 76 cells transduced with mo5 Vγ7Vδ2-2 TCR and co-cultured for 5 h with MODE-K.FLAG-l1.HA-l6 cells in the presence of the indicated concentrations of antibodies (x axis). Results were normalized to those obtained by co-culture with transduced MODE-K.EV cells. Data are representative of three independent experiments (mean ± SD of n = 3 co-cultures). (C) Specific staining of anti-His antibody alone (top row), soluble Vγ7^+^ TCR and anti-His mAb (middle row), or Vγ4^+^ TCR and anti-His mAb (bottom row) to 293T cells expressing Btnl1.6, BTNL3.8, or control 293T.EV. (D) Flow cytometry analysis of the staining of Btnl1.6-expressing 293T cells with increasing concentrations of soluble Vγ7^+^ TCR and anti-His mAb. See also Figure S2.

In addition, analogous results were obtained when Jurkat 76 (J76) cells expressing a mouse Vγ7 TCR were stimulated with MODE-K cells transduced with FLAG (N-terminal)-Btnl1 and HA (N-terminal)-Btnl6 constructs, in which case the anti-HA antibody more potently abrogated J76 activation (Figures 2A and 2B; Figure S2A). This was consistent with evidence that the responsiveness of Vγ7^+^ T cells to Btnl1.6 was more potently abrogated by mutations in the V region of Btnl6 compared with Btnl1, suggestive of Btnl6 being a direct ligand for Vγ7 (Melandri et al., 2018). Indeed, soluble Vγ7 TCR multimers specifically stained cells expressing Btnl1.6 (293T.l1l6) (Figure 2C; Figure S2B) in a dose-dependent manner (Figure 2D; Figure S2C), consistent with direct TCR-Btnl1.6 interactions.

Although our current and previous data suggested that multiple Vδ chains are compatible with Vγ4-mediated recognition of BTNL3.8, we could not exclude an effect of TCRδ. Compounding this, a recent study suggested that a certain Vγ4 TCR was not able to mediate BTNL3.8-driven responses in a cellular assay and suggested that particularly long Vδ1 CDR3 sequences might explain this observation (Mayassi et al., 2019). To address this more fully, we investigated Jurkat cells transduced with Vγ4 TCRs—either hu12-γ containing the H-J1 motif identified in active celiac disease IELs (Mayassi et al., 2019) or hu20-γ, a Vγ4 chain lacking the H-J1 motif (non-H-J1)—bearing a diverse range of Vδ1 chains (Melandri et al., 2018) (CDR3 ranged from 12 to 24 amino acids in length) for their capacity to upregulate CD69 and downregulate both TCR and CD3 in response to BTNL3.8-expressing cells. Conspicuously, all combinations resulted in similar degrees of γδ TCR and CD3 downregulation (Figure S2D). In addition, all constructs induced CD69 upregulation, although modest differences between different TCRs were observed, as we previously observed to be the case for TCR responses to anti-CD3 antibodies, as well as to BTNL- or Btnl-expressing cells (Melandri et al., 2018). These results establish that diverse Vδ1 CDR3 regions are permissive for Vγ4-mediated TCR triggering in response to recognition of BTNL3.8 on target cells.

### Germline-Encoded Regions of Vγ4 Dominate BTNL3 Interaction

**Figure 3:**
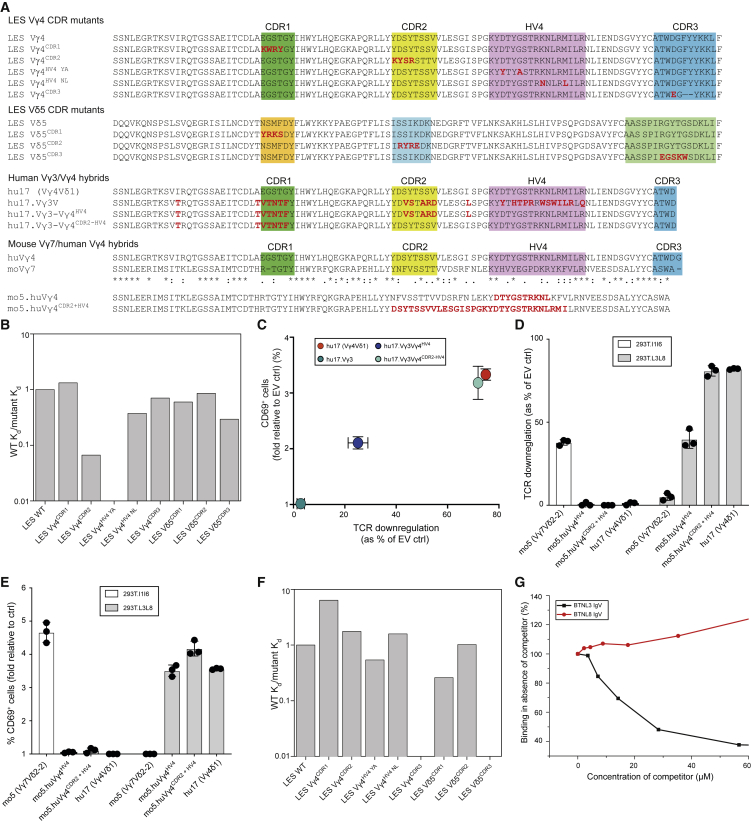
BTNL3 Binding to Vγ4 Involves Germline-Encoded Regions, whereas Antigen-Specific Binding Requires CDR3γ and CDR3δ Regions (A) Amino acid sequence of human Vγ4 and Vδ5 from the LES clone, showing mutations tested in CDR1, CDR2, HV4, and CDR3 in red below (top). (Middle) Amino acid sequence of human Vγ4 from the hu17 TCR aligned to human Vγ3 and the indicated Vγ3/hu17 Vγ4 hybrids transduced in JRT3. Red font indicates divergence from WT Vγ4. (Bottom) Alignment of the amino acid sequences of human Vγ4 with mouse Vγ7 and the CDR2 and HV4 chimeras generated in the mo5 Vγ7Vδ2-2 TCR. Red font indicates amino acids from human Vγ4 inserted in mouse Vγ7 to generate the indicated chimeras, expressed in Jurkat 76. (B) Binding affinity of BTNL3 to indicated Vγ4 and Vδ5 mutants (mutant K_d_) relative to WT LES TCR affinity (WT K_d_) measured in the same experiment. The averages of n = 4–5 experiments per Vγ4 mutant and 1–2 experiments per Vδ5 mutant are shown. (C) Flow cytometry analysis of TCR downregulation (x axis) plotted against that of CD69 upregulation (y axis) on JRT3 cells transduced with wild-type (WT) Vγ4Vδ1 TCR or the indicated Vγ3 or Vγ4 TCR hybrids and co-cultured for 4 h with 293T.L3L8 cells. Results were normalized to those obtained by co-culture with 293T.EV cells. Data are representative of two independent experiments (mean ± SD of n = 3 co-cultures). (D and E) Flow cytometry analysis of TCR downregulation (D) and CD69 upregulation (E) on J76 cells transduced with mo5 (Vγ7Vδ2-2) TCR or the indicated moVγ7/huVγ4 hybrid TCRs after co-culture with 293T.l1l6 or 293T.L3L8 cells. Results were normalized to those obtained by co-culture with 293T.EV cells. Data are representative of three independent experiments (mean ± SD of n = 3 co-cultures). (F) Binding affinity of EPCR to Vγ4 and Vδ5 mutants (mutant K_d_) relative to WT LES TCR affinity (WT K_d_) measured in the same experiment, representative of 2–3 experiments. (G) EPCR (3,012 RU) or control protein (2,586 RU) were immobilized on the sensor chip. WT LES TCR was injected over the surface at 12.5 μM in the presence of increasing specific competitor (BTNL3 IgV) or non-specific competitor (BTNL8 IgV). Binding responses were measured and are shown as a percentage of binding observed in the absence of competitor. See also Figure S3.

We then assessed which regions of the Vγ4 TCR chain were involved in directly engaging BTNL3. First, we generated Vγ4 TCRs with charge reversal mutations in each of the three CDRγ and CDRδ loops (Figure 3A). Second, based on amino acid sequence comparisons of Vγ4 with Vγ2 (which does not bind BTNL3), we generated Vγ4 TCRs incorporating mutations in the HV4γ loop, which we previously implicated in BTNL3-mediated triggering of Vγ4^+^ T cells (Melandri et al., 2018). We chose the LES Vγ4Vδ5 TCR as a model clonotype for these experiments, allowing comparison of the BTNL3 binding mode with that of endothelial protein C receptor (EPCR), which we previously identified as a unique ligand for the LES TCR (Willcox et al., 2012). BTNL3 binding affinity was substantially decreased by mutation of CDR2γ (Figure 3B; Figure S3A). Binding was even more strongly affected by substitution of Vγ2 residues Y/A into the Vγ4 HV4 loop (Figure 3B; Figure S3B), whereas substitution of N/L residues resulted in only marginally reduced affinity (Figure 3B). Consistent with this, LES TCR incorporating Vγ2 residues Y/A in the Vγ4 HV4 loop showed drastically reduced binding to BTNL3 by ITC (Figures S3C and S3D). Finally, compared with changes in the CDR2γ and HV4γ loops, mutations in CDR1γ, CDR3γ, CDR1δ, CDR2δ, and CDR3δ had either negligible or only modest effects on the interaction (Figure 3B). All soluble TCR (sTCR) mutants were folded correctly, as determined by pan-γδ TCR antibody binding (Figure S3E).

Based on these results, we hypothesized that the BTNL3-Vγ4 interaction was heavily focused on the CDR2 and HV4 loop regions of Vγ4. To test this, we generated chimeric constructs (Figure 3A) in which the CDR2 and/or HV4 loop regions of human Vγ4 replaced the counterpart regions of Vγ3 that are relatively divergent from Vγ4, differing overall by 24 amino acids, and then tested the ability of Jurkat cells transduced with those TCRs to upregulate CD69 and downregulate TCR in response to BTNL3.8-expressing cells. In support of our hypothesis, transductants expressing a chimeric Vγ3 construct that incorporated Vγ4 HV4 and CDR2 loops displayed degrees of CD69 upregulation and TCR downmodulation comparable to those of WT Vγ4 TCR transductants, whereas replacement of the HV4 loop alone conferred only partial recovery of reactivity upon BTNL3.8 (Figure 3C; Figure S3F).

In parallel, we generated constructs of a mouse Btnl1.6-reactive Vγ7 TCR in which the CDR2 and HV4 loops were replaced with their counterparts from human BTNL3.8-reactive Vγ4 (Figure 3A) and thereupon assessed the responsiveness of Jurkat transductants to mouse Btnl1.6 and human BTNL3.8, respectively. Introduction of either HV4 or a combination of HV4 and CDR2 sequences from human Vγ4 into the mouse Vγ7 sequence abolished murine Btnl1.6-mediated TCR downmodulation and CD69 upregulation yet concomitantly conferred reactivity to the human BTNL3.8 heterodimer (Figures 3D and 3E; Figure S3G). For TCR downmodulation, replacement of HV4 and CDR2 conferred ∼90% of the BTNL3.8-induced TCR downmodulation observed with WT Vγ4 TCR transductants, while HV4 replacement alone conferred a partial response. Of note, both the HV4 and the HV4 and CDR2 replacement constructs induced similar (>3-fold) increases in CD69 expression over control in response to BTNL3.8-expressing target cells. Altogether, these results confirm that reactivity to human BTNL3.8 is encoded substantially within the germline-encoded HV4 and CDR2 regions of human Vγ4 and that those regions alone were sufficient to convert a heterologous mouse TCR into one with almost full reactivity toward human BTNL3.8.

As a comparison for the BTNL3-TCR interaction, we tested how mutations in the CDR loops of the LES TCR (Figure 3A) affected EPCR binding. Mutation of LES CDR3γ or CDR3δ, which minimally affected BTNL3 binding, eliminated LES binding to EPCR (Figure 3F; Figures S3H and S3I), consistent with the pronounced and highly focused expansion of the LES clonotype observed following cytomegalovirus (CMV) infection (Lafarge et al., 2005). By contrast, mutation of other CDR loops in the Vγ4 or Vδ5 chains or of the HV4 region of Vγ4 only modestly affected LES binding to EPCR (Figure 3F). Thus, BTNL3 and EPCR bound Vγ4 via fundamentally distinct binding modes, raising the question of whether they could bind the LES TCR simultaneously. Arguing against this, however, co-incubation of the LES TCR with BTNL3 IgV decreased its EPCR binding in a concentration-dependent manner (to <40%). This inhibition was specific, as judged by the failure of co-incubation with BTNL8 IgV to affect EPCR binding (Figure 3G).

### Vγ4 TCR Interaction Involves the CFG Face of the BTNL3 IgV Domain

**Figure 4:**
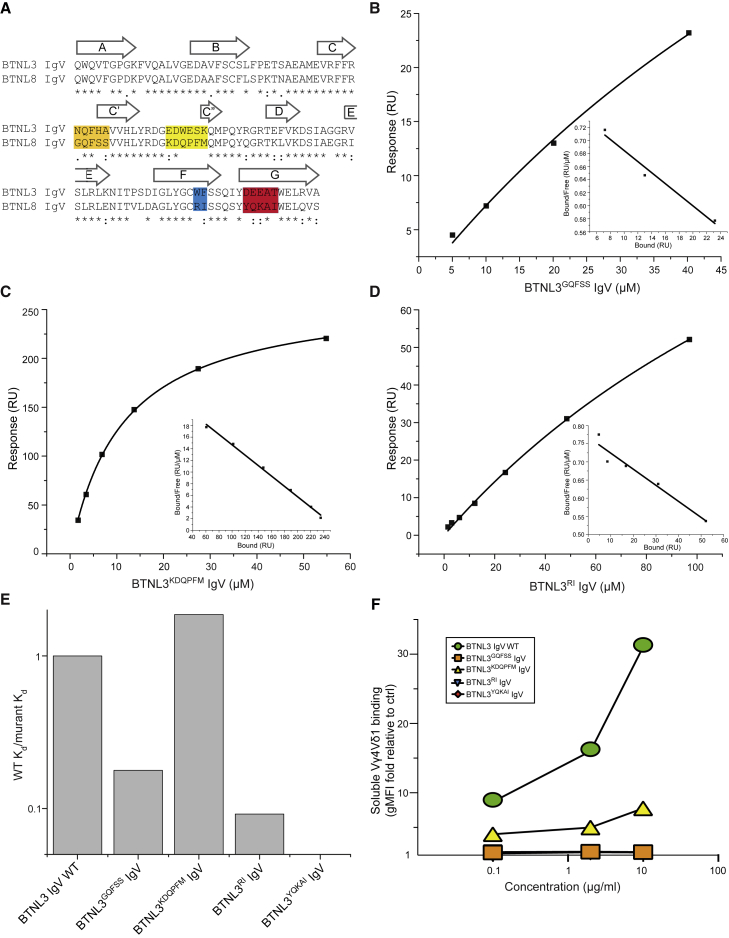
Regions of BTNL3 IgV Involved in Vγ4 Binding (A) Alignment of BTNL3 and BTNL8 IgV domains showing mutants generated. (B–D) Equilibrium affinity analysis of the binding of (B) BTNL3^GQFSS^ IgV (K_d_ = 117.6 μM), (C) BTNL3^KDQPFM^ mutant (K_d_ = 11.2 μM), or (D) BTNL3^RI^ mutant (K_d_ = 217.3 μM) to Vγ4 TCR. (E) Binding affinity of indicated mutants of BTNL3 (mutant K_d_) relative to WT LES TCR affinity (WT K_d_) measured in the same experiment. Data are representative of two experiments. (F) Flow cytometry analysis of 293T cells co-transduced with BTNL3 variants (as shown in the legend) and BTNL8 and stained with increasing concentrations (x axis) of soluble Vγ4Vδ1 and anti-His mAb. Results are presented as geometric mean fluorescence intensity (gMFI) of staining with the sTCR plus antibody to the His tag, normalized to the staining of 293T.EV cells under the same conditions. See also Figure S4.

To assess the regions of the BTNL3 IgV domain involved in binding, we used SPR to test the interaction with Vγ4 TCR of four BTNL3 IgV domain mutants that incorporated changes in the C′, C″ F, and G β strands of the BTNL3 IgV domain (CFG face), as was recently considered (Melandri et al., 2018) (Figure 4A). Upon injection, the BTNL3^GQFSS^, BTNL3^RI^, and BTNL3^YQKAI^ mutants each exhibited minimal specific binding to Vγ4 TCR, with a substantially reduced affinity relative to WT BTNL3 IgV, whereas the BTNL3^KDQPFM^ mutant clearly retained Vγ4 binding (Figures 4B–4E; Figures S4A–S4D). Moreover, relative to WT BTNL3, the BTNL3^RI^ mutant showed reduced binding affinity to Vγ4 TCR in ITC (K_d_ = ∼50 versus 3.5 μM for WT BTNL3) (Figures S4E and S4F). All BTNL3 mutants bound polyclonal anti-BTNL3 antibody (Figures S4G and S4H), suggesting that mutations in the CFG face did not inhibit expression or refolding of BTNL3 IgV. In parallel, we expressed the corresponding mutations as BTNL3.8 heterodimers in 293T cells and tested sTCR staining at the cell surface. Consistent with the SPR results, cells expressing BTNL3^GQFSS^.8, BTNL3^RI^.8, and BTNL3^YQKAI^.8 were not bound by sTCR, whereas the BTNL3^KDQPFM^.L8 mutant retained TCR staining, albeit reduced compared with WT BTNL3.8 (Figure 4F). Altogether, these experiments clearly implicate residues in the CFG face of BTNL3 in direct binding to the Vγ4 TCR chain. In addition, Vγ7 sTCR multimer staining to cells expressing Btnl1.6 was abolished by mutations of the counterpart positions of Btnl6 IgV (Melandri et al., 2018) (Figure S4I). Thus, a comparable mode of interaction is seemingly conserved from mouse through to human.

Although we did not directly address the role of the IgC domains of BTNL3 or BTNL8 in Vγ4 TCR recognition, homology modeling of the heterodimer based on the BTN3A1 homodimer (PDB: 4F80) confirmed a strong potential for BTNL3 IgC-BTNL8 IgC interchain heterodimer interactions (Figure S4J). Conversely, the formation of BTNL3.8 heterodimers analogous to non-symmetrical head-to-tail heterodimer interfaces observed in the crystal structure of BTN3A1, BTN3A2, and BTN3A3 (PDB: 4F80, 4F8Q, 4F8T) seems unlikely given comparisons of equivalent amino acids in BTNL3 IgV and BTNL8 IgC and in BTNL3 IgC and BTNL8 IgV (Figure S4K). These analyses suggest the BTNL3.8 heterodimer likely adopts a V-shaped dimer configuration similar to that observed in the BTN3A1 crystal lattice (Palakodeti et al., 2012), with the V region of one chain, BTNL3, available for direct binding to the CDR2-HV4 face of Vγ4.

### Parallels between BTNL3 Recognition and BTN3A1-Mediated P-Ag Sensing

**Figure 5:**
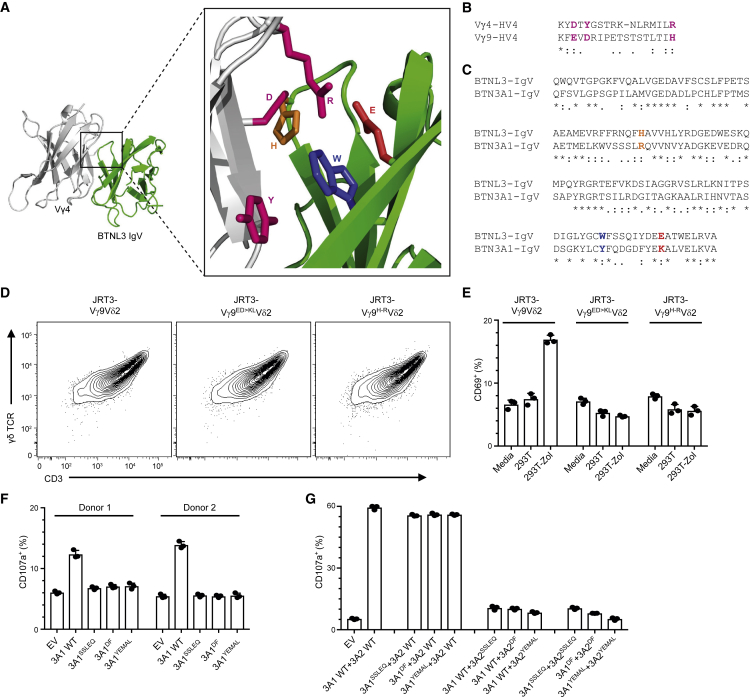
The HV4 Region of the Vγ9 TCR and the CFG Face of BTN3A1 and BTN3A2 Are Involved in the Phosphoantigen-Induced Activation of Vγ9Vδ2 T Cells (A) Best fit hypothetical model for docking of the Vγ4 TCR V domain (light gray) onto the BTNL3 IgV domain (green) (Melandri et al., 2018), generated using the computational docking program SwarmDock. Side chains are displayed for amino acids potentially directly involved in the contact between the Vγ4 HV4 region (D, Y, and R; pink) and the CFG face of the BTNL3-IgV domain (H61, W115, and E124; orange, blue, and red, respectively). (B and C) Alignment of the HV4 region of the Vγ4 and Vγ9 TCR V domains (B) and the IgV domains of BTNL3 and BTN3A1 and BTN3A2 (C). Amino acids of interest are colored as in (A). (D) Flow cytometry analysis of the expression of the indicated Vγ9Vδ2 TCR variants by JRT3 cells, 72 h post-transduction. (E) Flow cytometry analysis of CD69 upregulation by JRT3 cells expressing the indicated Vγ9Vδ2 TCR variants, following incubation with media only, or 293T cells with or without pre-treatment with zoledronate (Zol, 10 μM). Data are representative of three experiments (mean ± SD of n = 3 co-cultures). (F) Flow cytometry analysis of CD107a upregulation by polyclonal Vγ9Vδ2 T cell lines derived from peripheral blood mononuclear cells from two donors following co-culture with CRA123 cells transfected with the indicated BTN3A1 constructs (EV, empty vector control) and pre-treated with 10 μM Zol. Data are the mean ± SD of n = 3 co-cultures for each donor. (G) Flow cytometry analysis of CD107a upregulation by a polyclonal Vγ9Vδ2 T cell line following co-culture with CRA123 cells co-transfected with the indicated BTN3A1 + BTN3A2 constructs (EV, empty vector control) and pre-treated with 10 μM Zol. Data are the mean ± SD of n = 3 co-cultures. See also Figure S5.

To investigate whether the mode of ligand interaction employed by Vγ4 TCRs might have relevance for Vγ9-mediated P-Ag sensing, which is highly dependent upon the presence of BTN3A1 on target cells, we used a hypothetical model (Melandri et al., 2018) of the Vγ4 TCR-BTNL3 interaction we had previously generated with the computational docking program SwarmDock to deduce three pairs of amino acids likely to contribute directly to the interaction between Vγ4 and BTNL3. Vγ4-HV4 D, Y, and R were predicted to contact BTNL3 H, W, and E, respectively (Figure 5A). We then identified counterpart amino acids in Vγ9-HV4 (E, D, and H) by aligning with Vγ4-HV4 (Figure 5B; Figure S5A) and counterpart amino acids in BTN3A1-IgV (R, Y, and K) by aligning with BTNL3-IgV (Figure 5C; Figure S5B).

Next, we generated two HV4γ mutants, Vγ9^ED > KL^Vδ2 and Vγ9^H > R^Vδ2, which were designed to introduce charge alterations that might interfere with a putative analogous TCR-ligand interaction. Both mutants displayed efficient cell surface expression in JRT3 cells, equivalent to the WT TCR from which they were derived (Figure 5D), but abrogated CD69 upregulation in response to zoledronate (Zol)-treated 293T cells (Figure 5E; Figure S5C). Hence, P-Ag sensing by the Vγ9Vδ2 TCR was critically affected by specific HV4γ residues in counterpart positions to the Vγ4 residues that mediate BTNL3 interactions.

We then tested whether P-Ag sensing might likewise be affected by mutation of residues in the CFG face of the BTN3A1 IgV domain (SSLRQ, YF, and YEKAL) corresponding to those (NQFHA, WF, and DEEAT) implicated in the BTNL3 interaction with Vγ4 HV4 (Melandri et al., 2018, and as described earlier). Thus, we introduced non-conservative mutations of a single amino acid in each BTN3A1 motif (SSLRQ > SSLEQ, YF > DF, and YEKAL > YEMAL). When expressed in BTN3^−/−^ CRA123 cells (Vantourout et al., 2018), the 3A1^SSLEQ^ and 3A1^YEMAL^ mutants each displayed cell surface expression similar to that of WT BTN3A1 (Figure S5D), but compared with WT, they both failed to support the potential of Zol-treated CRA123 cells to stimulate two polyclonal Vγ9Vδ2 T cell lines (Figure 5F; Figure S5E). These findings clearly implicated the CFG face of BTN3A1-IgV in the activation of P-Ag-reactive Vγ9Vδ2 T cells, akin to the involvement of the counterpart regions of BTNL3 in provoking human Vγ4 responses (as described earlier). Note that although the 3A1^DF^ mutant also failed to support the potential of Zol-treated CRA123 cells to stimulate Vγ9Vδ2 cells, it could not be excluded that this was because this construct reached the cell surface inefficiently (Figure 5F; Figure S5D), thereby implicating the YF motif in the stringent regulation of BTN3A1 cell surface expression, as was previously considered (Vantourout et al., 2018).

Although BTN3A1 can be sufficient to support P-Ag stimulation of Vγ9Vδ2 T cells, this is greatly increased by co-expression of BTN3A2, which regulates the trafficking and cell surface expression of BTN3A1 via heteromerization (Vantourout et al., 2018). We therefore investigated whether the CFG face mutations of BTN3A1 could be complemented by WT BTN3A2, and vice versa*.* Thus, we generated the same CFG motif mutants of BTN3A2 and tested the expression of each combination (i.e., 3A1^mutant^ + 3A2^wt^, 3A1^wt^ + 3A2^mutant^, and 3A1^mutant^ + 3A2^mutant^) in CRA123 cells. Again, the SSLEQ and YEMAL mutants displayed cell surface expression similar to WT BTN3A1+BTN3A2, while the DF mutants showed decreased expression, particularly when both BTN3A1 and BTN3A2 carried this mutation (Figure S5F). However, when co-expressed with BTN3A2, the CFG face mutations in BTN3A1 negligibly affected Zol-induced CD107a upregulation by polyclonal Vγ9Vδ2 T cells (Figure 5G). By contrast, CFG face mutations in BTN3A2 could not be rescued by co-expression of WT BTN3A1, impairing CD69 upregulation almost as much as when BTN3A1 and BTN3A2 were both mutated in the CFG faces (Figure 5G; Figure S5G). Moreover, similar impacts were observed when the responders were JRT3 cells expressing a WT Vγ9Vδ2 TCR (Figures S5H and S5I). Altogether, these data show that three systems of BTN-mediated γδ T cell regulation—human Vγ4 and BTNL3.8, murine Vγ7 and Btnl1.6, and human Vγ9 and BTN3A—receive critical contributions from HV4 of the relevant TCR Vγ chain and from specific, orthologous IgV-CFG face residues of the relevant BTNs.

## Discussion

Over the past decade, it has become clear that BTN and BTNL/Btnl members of the B7 superfamily play critical roles in γδ T cell selection and activation in mice and humans, spanning both peripheral blood and tissue-associated subsets (Barbee et al., 2011, Boyden et al., 2008, Di Marco Barros et al., 2016, Harly et al., 2012, Turchinovich and Hayday, 2011). Nonetheless, although several studies have shed light on the mechanism(s) underpinning this profound biology, key aspects have remained largely unresolved, in particular whether there are direct interactions of the relevant TCRs with BTN, BTNL, or Btnl proteins. Addressing this, the current study provides unequivocal evidence that the human BTNL3.8 heterodimer interacts directly with Vγ4 γδ TCRs, specifically via the BTNL3 chain. This builds on previous work demonstrating that cells expressing BTNL3.8 complexes could induce TCR-mediated stimulation of human Vγ4 gut T cells, while the counterpart murine molecules, Btnl1.6, induced TCR triggering of mouse gut Vγ7 T cells (Di Marco Barros et al., 2016, Melandri et al., 2018).

Previously, the only evidence of BTN or BTNL directly engaging the TCR was provided by Vavassori et al. (2013), who reported direct binding of a Vγ9Vδ2 TCR to the BTN3A1 ectodomain, which was also reported to present P-Ag. However, both findings have been convincingly challenged (Sandstrom et al., 2014), and our laboratory has similarly failed to reproduce Vγ9Vδ2 TCR-BTN3A1 binding. In contrast to this, the current study, which incorporates evidence from a combination of SPR, ITC, and mutagenesis, not only documents direct binding of the BTNL3 IgV domain to Vγ4 but also demonstrates an interaction modality resembling that of superantigens (Sundberg et al., 2007) in its complete dependence on germline-encoded Vγ4 HV4 and its substantial reliance on germline-encoded Vγ4 CDR2.

Although our study focused predominantly on the LES clonotype as a model Vγ4 TCR, the binding modality we outline fully explains both the specificity of BTNL3 binding to Vγ4 TCRs, but not to Vγ2 or Vγ3 TCRs, and our previous findings that BTNL3.8 dimers trigger TCR downregulation of essentially any TCRs that are Vγ4^+^, irrespective of CDR3γ or TCR Vδ usage (Melandri et al., 2018). In this respect, it is challenging to understand the claim that a Vγ4^+^ TCR identified in a celiac disease gut failed to respond to BTNL3-expressing cells (Mayassi et al., 2019). Indeed, by using a cellular assay of TCR and CD3 downregulation, we show here that manipulating the length and sequence of the Vδ1-CDR3 region had negligible, if any, effects on the efficiency of BTNL3.8 recognition, even when long CDR3 regions were incorporated. Despite this, some modest differences in CD69 upregulation were observed. The failure of Mayassi et al. (2019) to detect a BTNL3-mediated response of a particular Vγ4^+^ TCR might reflect their use of a less sensitive assay system. This notwithstanding, we cannot formally exclude that rare CDR3δ regions might indirectly affect the interaction, for example, via effects on CDR2 loop conformation. Indeed, we note that different TCRs can transduce quantitatively different signals in response to anti-CD3 agonist antibodies (Melandri et al., 2018), which must also reflect indirect effects.

Considering that BTNL3.8 and Btnl1.6 heteromers are expressed specifically by intestinal epithelial cells of humans and mice, respectively, extrapolation of the findings presented here to Btnl6-mediated interactions with Vγ7 would readily explain how BTNL/Btnl proteins can act as tissue-specific, non-clonal selecting elements for signature γδ T cell compartments defined by discrete Vγ chains. Our demonstration in the current study that mouse Vγ7 multimers specifically stain Btnl1.6-expressing target cells, combined with mutagenesis studies, is consistent with the evolutionary conservation of the mode of action to BTNL3.8.

By focusing on the LES Vγ4 TCR that we have previously shown to recognize EPCR (Willcox et al., 2012), we were able to compare TCR recognition of BTNL3 with that of a clonally restricted antigenic ligand. The private LES clonotype expanded substantially in an individual after CMV infection, adopted an effector phenotype (Lafarge et al., 2005), and appeared to align closely with the adaptive biology that has recently emerged for some human γδ T cell subsets, including Vδ2^neg^ and Vγ9^neg^Vδ2^+^ T cells, which can both demonstrate highly focused clonotypic expansion and differentiation after antigenic challenge (Davey et al., 2018a). Our study provides unequivocal data that BTNL3 engages the TCR by a qualitatively different modality to that by which clonally restricted ligands engage the TCR. Whereas the former (BTNL3) modality focused predominantly on germline-encoded regions (the HV4 and CDR2 regions were sufficient to confer essentially complete BTNL3.8 recognition upon human Vγ3 and upon a heterologous mouse Vγ7 TCR), the latter (EPCR) modality was energetically focused on CDR3γ and CDR3δ regions that are generated by somatic gene rearrangement. Although this latter CDR3 mode might be expected to incur substantial entropic penalties upon binding, as for both CDR3-driven antibody-antigen and αβ TCR-peptide-major histocompatibility complex (MHC) binding (Stites, 1997, Willcox et al., 1999), a notable feature of Vγ4^+^ TCR-BTNL3 interaction evident from our ITC data was a relatively modest change in entropy, suggestive of a more rigid body interaction mode. This is consistent with our hypothetical model of Vγ4^+^ TCR-BTNL3 interaction (Melandri et al., 2018), whereby the HV4 and CDR2 elements involved in the Vγ4^+^ TCR derive from β sheet structure, which might remain relatively rigid even in the unbound state. Future studies will be needed to understand whether this is a common feature of TCR-BTN, TCR-BTNL, and mouse TCR-Btnl protein interactions.

The full scope of these two ligand interaction modalities is currently unclear. Possibly, one modality is exclusive of the other; consistent with this, our results indicate the LES TCR is unable to engage both BTNL3 and EPCR synchronously. This argues strongly against any universal requirement for co-engagement of BTNL3 and antigenic ligands in TCR-mediated stimulation of Vγ4^+^ T cells. Consistent with this, in some donors, clonally expanded Vγ4Vδ1^+^ and Vγ4Vδ2^+^ adaptive-like populations can be identified in both peripheral blood and liver where BTNL3.8 is not expressed (Davey et al., 2017, Hunter et al., 2018, Di Marco Barros et al., 2016). However, it remains possible that there is sequential use of the two modalities: thus, selection and/or tonic engagement of BTNL3.8 may be an essential preface to clonotypic antigen engagement within the Vγ4^+^ T cell compartment in response to various forms of tissue dysregulation.

Alternatively, it cannot be discounted that one γδ T cell might only ever respond via one of the two modalities. By this means, γδ T cells would collectively provide complementary, innate-like, and adaptive arms to the γδ T cell response. Studies on adaptive γδ T cells have highlighted their potential, following antigenic challenge, to populate tissues with clonally expanded subsets that have heightened effector capability and are likely to provide ongoing immune surveillance against recurrently encountered challenges (Hunter et al., 2018). However, such responses are likely to be highly dependent upon exposure to specific immunological scenarios such as pathogen infection, including CMV (Davey et al., 2017, Davey et al., 2018b, Ravens et al., 2017), or possibly antigenic changes underlying autoimmune or inflammatory conditions.

In contrast, the constitutive, selective tissue associations of BTNL/Btnl molecules have the potential to pre-populate relevant tissues with functionally distinct innate-like γδ T cells (Boyden et al., 2008, Di Marco Barros et al., 2016, Turchinovich and Hayday, 2011). Given that the respective TCR binding modalities we observe might permit γδ T cells collectively to make either innate-like or adaptive responses, it will be important to study whether the two modalities induce distinct TCR-mediated signaling pathways, as seems to be the case for αβ TCR-activating superantigens that engage HV4 regions of TCR Vβ versus peptide-MHC engagement that depends on the CDR3 regions (Bueno et al., 2006). Studies on intraepithelial γδ T cell populations have suggested that these cells can make growth factors such as keratinocyte growth factor, insulin-like growth factor, and amphiregulin, consistent with roles in epithelial homeostasis (Boismenu and Havran, 1994, Krishnan et al., 2018, Mayassi et al., 2019, Shires et al., 2001). Conceivably, engagement of BTNL/Btnl family members (or related Skint molecules in the skin) might stimulate the cells’ production of growth factors involved in tissue repair and homeostasis, while engagement of clonally restricted antigen might induce different functional outcomes, such as the production of interferon (IFN) γ, or interleukin-13 (IL-13) (Dalessandri et al., 2016). In this regard, the celiac gut was reported to harbor an enrichment of more adaptive, clonally expanded Vγ4^−^ γδ T cells, which were hypothesized to contribute to inflammation (Mayassi et al., 2019).

Future studies should explore the generality of bimodal antigen receptor binding. Several sets of data, including some presented here, strongly argue that this will be conserved for murine intestinal intraepithelial Vγ7 cells, which are strictly regulated by Btnl1.6 heteromer but may display CDR3-mediated clonal or oligoclonal responses to other antigens, including T10 and T22 (Shin et al., 2005). In particular, our observation that Vγ7 TCR-Btnl1.6 recognition is abolished either by substituting mouse Vγ7 CDR2 and HV4 regions with those of human Vγ4 or by mutating Btnl6 residues at positions equivalent to BTNL3 residues implicated in Vγ4 TCR binding strongly suggests a Vγ7-Btnl6 recognition mode analogous to Vγ4-BTNL3.

Likewise, although BTN3A1-dependent P-Ag responses of human peripheral blood Vγ9Vδ2 cells are irrefutably associated with specific TCRγ junction (Jγ) region sequences (Bukowski et al., 1998, Delfau et al., 1992), in the manner of adaptive reactivities, the rapid and universal response of Vγ9Vδ2 cells to P-Ags (Davey et al., 2018b, Morita et al., 1995) appears to be innate-like. Despite the highly distinct biology of Vγ9Vδ2 lymphocytes relative to intestinal Vγ4^+^ and Vγ7^+^ T cells, it is striking that their BTN3A-dependent response to P-Ags was eliminated by Vγ9 HV4 mutations in counterpart positions to Vγ4 HV4 mutations that abrogate BTNL3 binding and that BTN3A-CFG mutations analogous to BTNL3 regions involved in binding Vγ4 severely diminished P-Ag sensing. In addition, the strong impact of mutations in BTN3A2 relative to BTN3A1 was highly intriguing, particularly given their identical IgV domain sequence. Clearly, additional work is needed to clarify the precise molecular targets recognized by the Vγ9Vδ2 TCR and the modalities by which they are engaged. Data presented here further implicate the V region of BTN3A2 in Vγ9Vδ2 T cell responses, which likewise merits follow-up. Importantly, in light of the failure to detect direct binding of Vγ9Vδ2 TCR to BTN3A1, our mutational data might imply that BTN3A1 or BTN3A2 does indeed interact with the Vγ9Vδ2 TCR but either does so weakly and/or in concert with additional moieties, candidates for which might include the F_1_-ATPase (Scotet et al., 2005). Supporting the feasibility of this suggestion, a recent crystallographic study of pollen allergen-antibody recognition highlighted simultaneous interaction of each Fab fragment with two separate ligand surfaces: one via a superantigen-like modality involving germline-encoded receptor regions and the other involving a more conventional CDR3-mediated interaction (Mitropoulou et al., 2018).

In the most extreme generality, the existence of distinct, parallel ligand recognition modes might be a feature of many antigen receptors. Thus, there are αβ TCRs that can engage endogenous or exogenous superantigens via germline-encoded subdomains and peptide-MHC complexes largely via recombination-dependent CDR3 subregions (Sundberg et al., 2007). Moreover, there is increasing interest in antibodies that disproportionately employ HV4 in antigen binding (Meyer et al., 2016). In this regard, our present and prior studies argue strongly that the γδ TCR retains the potential for bimodal ligand recognition. Moreover, this appears to be an evolutionarily conserved trait, at least from mice to humans, with many properties of mouse Vγ7 being shared with human Vγ4. Given that distinct tissue associations based on Vγ region usage are a signature of several γδ T cell populations in mice, it may be that other B7-like ligands operate at defined anatomical sites to engage the HV4 and CDR2 regions of the relevant murine TCR Vγ regions, thereby regulating unique, tissue-specific subsets of γδ T cells. In this regard, the emerging alignment between mouse and human γδ T cell biology in TCR-mediated recognition of BTNL molecules fuels optimism that further studies on mouse γδ TCR ligands may greatly inform human γδ T cell biology and its relationship to myriad pathophysiologies. Furthermore, given that HV4 and CDR2 are germline-encoded, their interactions with ligands may have contributed to the inheritance patterns of particular V gene segments across Ig, αβ TCRs, and γδ TCRs.

## STAR★Methods

### Key Resources Table

**Table:** 

REAGENT or RESOURCE	SOURCE	IDENTIFIER
**Antibodies**		

Vγ7-AF647 (clone F2.67)	P. Peirera, Institut Pasteur, Paris, France	N.A.
γδTCR-PerCPeFluor710 (clone GL3)	Invitrogen	Cat#46-5711-82; LOT 4324311; RRID: AB_2016638
γδTCR-APC (clone GL3)	Biolegend	Cat#118116; LOT B228498; RRID: AB_1731813
CD69-PE (clone FN50)	Biolegend	Cat#310906; LOT B258744; RRID: AB_314840
CD3-PerCPCy5.5 (clone SK7)	Biolegend	Cat#344808; LOT B253485; RRID: AB_10641704
CD3-BV421 (clone SK7)	Biolegend	Cat#344833; LOT B250131; RRID: AB_2565674
His-tag-APC (clone J095G46)	Biolegend	Cat#362605; LOT B250305; RRID: AB_2715818
FLAG (unlabeled) (clone L5)	Biolegend	Cat#637302; LOT B185582; RRID: AB_1134268
FLAG-PE (clone L5)	Biolegend	Cat#637310; LOT B182164; RRID: AB_2563148
FLAG-APC (clone L5)	Biolegend	Cat#637308; LOT B182164; RRID: AB_2561497
HA (unlabelled) (clone 16B12)	Biolegend	Cat#901502; LOT B242905; RRID: AB_2565007
HA-AF647 (clone 16B12)	Biolegend	Cat#682404; LOT B246404; RRID: AB_2566616
Mouse IgG1 isotype control (clone MOPC-1)	Biolegend	Cat#400166; LOT B230982; RRID: AB_11146992
CD3-BV421 (clone OKT3)	Biolegend	Cat#317344; LOT 248594; RRID: AB_2565849
CD3-AF647 (clone OKT3)	Biolegend	Cat#317312; LOT B224782; RRID: AB_571883
CD69-AF647 (clone FN50)	Biolegend	Cat#310918; LOT B246313; RRID: AB_528871
γδTCR-PECy7 (clone IMMU510)	Beckman Coulter	Cat#B10247; LOT 33
TCRVδ2-FITC (clone B6)	Biolegend	Cat#331406; LOT B224768; RRID: AB_1089230
CD107a-PE (clone H4A3)	Biolegend	Cat#328608 ; LOT B264921; RRID: AB_1186040
Purified mouse anti-human TCRγ/δ, clone 11F2	BD Biosciences	Cat# 347900; RRID: AB_400356
BTNL3 (unlabelled, rabbit polyclonal)	Aviva Systems Biology	Cat#ARP46769_P050; RRID: AB_2045124

**Bacterial and Virus Strains**		

Stbl2	ThermoFisher	Cat#10268019
NEB 5-alpha	NEB	Cat#C2987H
BL21 (DE3)	NEB	Cat#C2527H

**Biological Samples**		

HMBPP expanded human Vγ9Vδ2+ T cells	Vantourout et al., 2018	N/A

**Chemicals, Peptides, and Recombinant Proteins**		

Soluble monomeric Vγ4Vδ1 TCR, His-tagged	GammaDelta Therapeutics	N/A
NdeI	Roche	Cat#11 040 227 001
BamHI	Roche	Cat#10 567 604 001
BTNL3 IgV	This paper	N/A
BTNL8 IgV	This paper	N/A
Soluble T cell receptors (sTCRs)	Willcox et al., 2012 and this paper	N/A
Soluble EPCR	Willcox et al., 2012 and this paper	N/A

**Experimental Models: Cell Lines**		

J76 (human)	Francis Crick Institute (FCI) Cell Services	N/A
MODE-K (mouse)	Gift from Dr. D. Kaiserlian, INSERM U1111, Lyon, France	N/A
JRT3 (human)	Gift from Dr. S. Mansour, University of Southampton	N/A
293T (human)	ATCC	N/A
293T BTNL variants and EV cell lines	Di Marco Barros et al., 2016, Vantourout et al., 2018, Melandri et al., 2018	N/A
MODE-K Btnl variants and EV cell lines	Di Marco Barros et al., 2016, Melandri et al., 2018	N/A
293T CRA123 (BTN3 locus deletion)	Vantourout et al., 2018	N/A
J76/JRT3 TCR variants	This paper and Melandri et al., 2018	N/A

**Oligonucleotides**		

Full length cloning primers for BTN3, Btnl, BTNL	Di Marco Barros et al., 2016, Vantourout et al., 2018, Melandri et al., 2018;	N/A

**Recombinant DNA**		

pCSIGPW, pCSIYHW	Di Marco Barros et al., 2016	N/A
pCR/V1	Zennou et al., 2004	N/A
pHIT/G	Fouchier et al., 1997	N/A
pET23a	Merck Millipore	Cat#69745-3
pMT/BiP/V5-HisB	Invitrogen	Cat#V413020
FLAG-Btnl1 in pCSIYHW	Di Marco Barros et al., 2016	N/A
HA-Btnl6 in pCSIGPW	Di Marco Barros et al., 2016	N/A
Human and mouse γδTCRs in pCSIGW	This paper and Melandri et al., 2018	N/A
FLAG-BTNL3 in pCSIGPW	This paper and Melandri et al., 2018	N/A
HA-BTNL3 in pCSIGPW	This paper and Melandri et al., 2018	N/A
FLAG-BTN3A1 in pCSIGPW	This paper and Vantourout et al., 2018	N/A
HA-BTN3A2 in pCSIGPW	This paper and Vantourout et al., 2018	N/A
BTNL3 IgV in pET23a (wild type and mutants)	This paper	N/A
BTNL8 IgV in pET23a	This paper	N/A
Human and mouse γδTCRs in pMT/BiP/V5-HisB	This paper and Willcox et al., 2012	N/A
EPCR in pMT/BiP/V5-HisB	Willcox et al., 2012	N/A

**Software and Algorithms**		

FlowJo version 10	FlowJo LLC	https://www.flowjo.com/
PyMOL version 2.0.7	Schrodinger LLC	https://pymol.org
GraphPad Prism versione 8.0.2	GraphPad Software LLC	https://www.graphpad.com
BIAevaluation	GE Healthcare	https://www.gelifesciences.com/en/gb/shop/protein-analysis/spr-label-free-analysis
Origin 2015	OriginLab	https://www.originlab.com/

**Other**		

Sensor Chip CM5	GE Healthcare	29149604
Sensor Chip NTA	GE Healthcare	BR100407
HBS-P	GE Healthcare	BR100368
HBS-EP	GE Healthcare	BR100188
Streptavidin	Sigma	S4622

### Lead Contact and Materials Availability

For additional information about reagents and resources, contact the lead contact, Benjamin E. Willcox, at b.willcox@bham.ac.uk.

### Method Details

#### Soluble Protein Production

cDNA encoding wt BTNL3 or BTNL8 IgV domains (Q18 to V131), or BTNL3 IgV incorporating the described mutations, were generated as gblocks (Integrated DNA Technologies) including the sequence for a C-terminal 6x Histidine tag, cloned into the pET23a expression vector (Novagen), and were overexpressed in *E. coli* BL21 (DE3) strain (NEB). Protein expression was induced by addition of 0.5mM isopropyl-b-D-1-thiogalactopyranoside and culture for 4 hours at 37°C. The bacterial cell pellet was harvested by centrifugation at 7500 g for 20 min, resuspended in PBS and lysed by mechanical disruption using an Emulsiflex C3. The overexpressed inclusion body protein was isolated by centrifugation at 44000 g for 30 min. The pellet was washed three times in 50mM Tris, pH8, 0.5% Triton X-100, 2 mM DTT, and 0.1% sodium azide, and once in 50 mM Tris, pH8, 2 mM DTT, and 0.1% sodium azide, then solubilised in 8 M urea, 50 mM MES, pH 6.5, 1 mM EDTA, 2 mM DTT. BTNL3 or BTNL8 inclusion body proteins were further reduced by addition of fresh 20 mM DTT for 30 min at 37°C immediately prior to refolding. BTNL3 IgV was refolded by dilution in 100 mM Tris, 400 mM L-Arginine-HCl, 2 mM EDTA, 6.8 mM cystamine, 2.7 mM cysteamine, 0.1 mM PMSF, pH 8, overnight at 4°C. BTNL8 IgV was refolded as described for Skint1 IgV (Salim et al., 2016). The refolding mixture was concentrated down and purified by size exclusion chromatography on a Superdex-200 column (GE Healthcare) pre-equilibrated with 20 mM Tris, 150 mM NaCl, pH 8, or PBS. Soluble γδ TCRs (LES TCR (Vγ4^+^; wt and mutants), Vγ2^+^ TCR, Vγ3^+^ TCR and mouse Vγ7^+^ TCR) and soluble EPCR were generated in *Drosophila* S2 cells and purified by nickel chromatography as previously described (Willcox et al., 2012). Mutant Vγ4 and Vδ5 constructs were generated using the Quickchange site-directed mutagenesis kit (Stratagene) of wt LES TCR constructs in pMT/BiP/HisB (Invitrogen) (Willcox et al., 2012). TCRs were then biotinylated via a C-terminal BirA tag. Soluble Vγ4Vδ1 TCRs used to stain 293T.L3L8 cells were described previously (Melandri et al., 2018).

#### Surface Plasmon Resonance

SPR was performed as described (Willcox et al., 1999) on a BIAcore3000 using streptavidin-coated CM5 chips and HBS-EP buffer (GE Healthcare). Biotinylated wt or mutant LES TCR and control Vγ2 or Vγ3 TCRs (1000-3500 RU), were captured on the Streptavidin chip. Analyte concentrations ranged from 1-200 μM. The BTNL3-EPCR competition assay was performed by immobilising 2500-3000 RU of wt EPCR or control protein (EPCR R127A mutant, which abrogated binding to LES TCR), (Willcox et al., 2012) on the surface of the chip. Injections of LES TCR at a constant concentration of 12.5 μM were performed in the presence of increasing concentrations of BTNL3 IgV (3.5-113 μM), or BTNL8 IgV (2.2-70.5 μM). Binding of BTNL3 polyclonal antibody was measured following immobilisation of His-tagged BTNL3 IgV mutants to an NTA Sensor Chip in HBS-P (GE Healthcare).

#### Isothermal Titration Calorimetry

Calorimetric measurements were carried out using an iTC200 instrument (Malvern Panalytical, Malvern, UK). All experiments were performed at 20°C, with proteins purified in 20mM Tris pH 8, 50 mM NaCl. Typically, TCR proteins were contained in the calorimeter cell at a concentration of 70 μM, into which BTNL3 proteins at a ten-fold higher molar concentration were titrated as 20 × 2 μl injections. All ITC binding experiments were corrected for heats of dilution. Binding data were analyzed by fitting the binding isotherm to a single independent binding site model using Origin software.

#### Cell Lines

293T and MODE-K cells were maintained in DMEM supplemented with 4.5 g/L D-glucose, L-glutamine, 10% heat-inactivated fetal calf serum (FCS) and 1% penicillin-streptomycin (Complete DMEM). JRT3 and Jurkat 76 (J76) were maintained in RPMI 1640 with L-glutamine, 10% heat-inactivated FCS and 1% penicillin-streptomycin (Complete RPMI). All cell culture reagents were from Thermo Fisher. Polyclonal Vγ9Vδ2 T cell lines were generated from PBMC from healthy donors after informed consent as described previously (Vantourout et al., 2018).

#### Reagents

Transduced 293T and MODE-K cells were grown in Complete DMEM supplemented with 1 μg/mL puromycin (Sigma-Aldrich) alone or in combination with 500 ng/mL hygromycin B (Thermo Fisher). Polyethylenimine was from Polysciences. Zoledronate (Zol) was from Sigma-Aldrich.

#### Mutagenesis and Molecular Biology

Plasmids encoding hu17 (human Vγ4Vδ1), huPB (human Vγ9Vδ2) and mo5 (murine Vγ7Vδ2-2) TCRs, and BTNL and Btnl variants, and BTN3A1 and BTN3A2 have been described (Melandri et al., 2018, Vantourout et al., 2018). Chimeric and mutant TCRs were generated using overlap-extension PCR (OE-PCR) and cloned into the self-inactivating lentiviral vector pCSIGPW after removal of the IRES-GFP and CMVp-Puro^R^ cassettes. OE-PCR was likewise used to mutate BTN3A1 and BTN3A2, which were subsequently cloned into pCSIGPW.

#### Lentiviral Transduction

Lentiviral particles were produced in wild-type 293T cells by co-transfection with lentiviral plasmids encoding target proteins (derived from pCSIGPW), HIV-1 *gag-pol* pCR/V1 (Zennou et al., 2004) and VSV-G *env* pHIT/G (Fouchier et al., 1997) using PEI. Medium was replaced after 16 h and collected 48 h post-transfection, filtered through 0.45 μm nylon mesh, and used to transduce target cell lines. JRT3/J76 cells were transduced by spinoculation at 1,000 *g*, 20°C for 30 min. 5x10^5^ 293T cells/well were plated in a 12-well plate a day prior to transduction. The following day, supernatants from the packaging cell lines were mixed 1:1 and 1.5 mL was used to transduce plated 293T cells. Culture medium was supplemented with antibiotics for selection 24 h post-transduction. Transductants were bulk-sorted on uniform GFP expression.

#### Co-culture Assay

0.5 × 10^5^ JRT3/J76 transductants or PBMC-derived polyclonal Vγ9Vδ2 T cells were mixed in 96-well plates with 1.5 × 10^5^ 293T or MODE-K cells, followed by co-culture for 5 h. 293T transiently expressing BTNL3 and BTNL8 (293T.L3L8) were used in blocking experiments. 48 h post-transfection, 293T cells were harvested and pre-incubated for 30 min at 4°C with α-FLAG, α-HA or IgG control antibodies (L5, 16B12, MG1-45 respectively; BioLegend). JRT3 were subsequently added and the cells were co-cultured for 3h at 37°C in the presence of the antibodies. For blocking experiments of murine Btnl molecules, MODE-K stably expressing Btnl1+Btnl6 (MODE-K.l1.l6) were pre-incubated for 60 min at 37°C with α-FLAG, α-HA or IgG control antibodies in 96-well plates. J76-mo5 were added to the wells and cells were co-cultured for 5h in the presence of the antibodies. 293T-CRA123 cells were transiently transfected with BTN3 constructs. Media was replaced 24 h post-transfection with Complete DMEM supplemented with Zol (10 μM). Cells were maintained for 16 h, washed twice, and co-cultured with JRT3 transductants or PBMC-derived polyclonal Vγ9Vδ2 T cell lines as described above.

#### Flow Cytometry

Flow cytometry was performed using the following antibodies from BioLegend, unless otherwise stated. Antibodies to the following human molecules were used: CD69-AF647 (FN50), CD69-PE (FN50), CD3-BV421 (OKT3), γδTCR-PeCy7 (IMMU510; Beckman Coulter), CD45-PacificBlue (HI30), TCRVδ2-FITC (B6). Antibodies to the following murine molecules were used: TCRδ-PerCPe710 (GL3), TCRδ-APC (GL3), Vγ7-AF647 (F2.67, provided by P.Pereira). Other antibodies were as follows: DYKDDDDK-PE (FLAG, L5), HA-AF647 (16B12). 6xHis-APC (Biolegend, J095G46). Data were acquired on BD Canto II or Fortessa cytometers. sTCR staining was performed as in Melandri et al. (2018).

#### Software

Flow cytometry data were analyzed in FlowJo (versions 9 and 10; FlowJo, LLC) and Prism (version 7; GraphPad). Structural figures were generated in PyMOL (version 2.0.7; Schrodinger, LLC). For SPR and ITC, data were analyzed in BIAevaluation (GE Healthcare) and Origin 2015 (OriginLab).

#### Molecular Modeling

Molecular models of the BTNL-3 and BTNL-8 IgV-IgC domains were derived from the I-TASSER server (Yang et al., 2015). BTNL-3 and BTNL-8 IgV-IgC domain models were then superimposed onto the equivalent regions of previously published BTN3A1 structure adopting either the V-shaped or head-to-tail dimer (Palakodeti et al., 2012). Residues that contribute to stabilizing BTN3A1 and BTNL-3/BTNL-8 dimer interfaces were identified using programs of the CCP4 suite (Winn et al., 2011).

### Quantification and Statistical Analysis

Flow cytometry data were analysed in FlowJo (versions 9 and 10; FlowJo) and Prism (version 7; GraphPad). Structural figures were generated in PyMOL (version 2.0.7; Schrodinger). For SPR and ITC, data were analysed in BIAevaluation (GE Healthcare) and Origin 2015 (OriginLab).

### Data and Code Availability

There is no data or availability to report.

## Supplemental Information

Document S1. Figures S1–S5

Document S2. Article plus Supplemental Information
