# Supplementary material for: Butyrophilin-like 3 Directly Binds a Human Vγ4+ T Cell Receptor Using a Modality Distinct from Clonally-Restricted Antigen
Source: Immunity. 2019 Nov 19;51(5):813–825.e4. doi: 10.1016/j.immuni.2019.09.006 (PMC6868513; doi:10.1016/j.immuni.2019.09.006)
Supplement: Document S1. Figures S1–S5 [file mmc1.pdf]

Immunity, Volume *51*

Supplemental Information

Butyrophilin-like 3 Directly Binds a Human Vγ4^{+} T Cell Receptor Using a Modality Distinct from Clonally-Restricted Antigen

Carrie R. Willcox, Pierre Vantourout, Mahboob Salim, Iva Zlatareva, Daisy Melandri, Leonor Zanardo, Roger George, Svend Kjaer, Mark Jeeves, Fiyaz Mohammed, Adrian C. Hayday, and Benjamin E. Willcox

Supplementary Figure 1

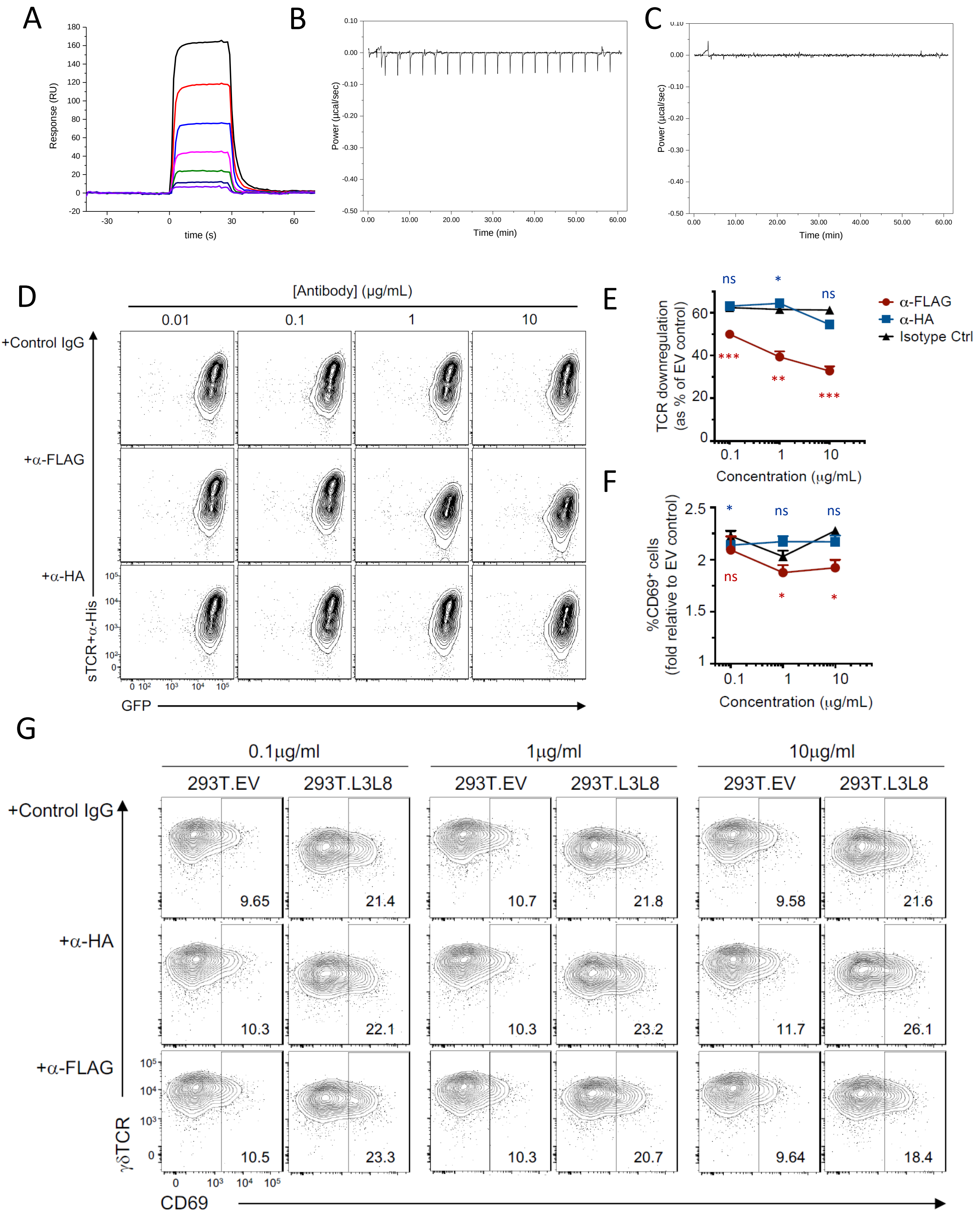

Supplementary Figure 1 - related to main text Figure 1. (A) Equilibrium binding of BTNL3 IgV (0.6-36 μM) to Vγ4 TCR immoblised on the sensor surface (1805 RU). Responses to a control TCR (1872 RU) have been subtracted. Raw ITC traces showing injection of BTNL3 IgV domain into solution containing Vγ2 TCR (B) or Vγ3 TCR (C). (D) Representative flow-cytometry analysis of soluble Vγ4Vδ1 TCR binding to 293T.FLAG-L3.HA-L8 cells (visualized by anti-His) following pre-incubation with indicated concentrations of anti-FLAG, anti-HA or IgG control antibodies (see Figure 1F). (E,F) Flow-cytometry analysis of TCR downregulation and CD69 upregulation by JRT3 cells transduced with hu17 Vγ4Vδ1 TCR and co-cultured for 3 h with transfected 293T.FLAG-L3.HA-L8 cells in the presence of the indicated concentrations of anti-FLAG (red) or anti-HA (blue) antibody; results were normalized to those obtained by co-culture with transfected 293T.EV cells in the presence of the same antibody concentrations. Data are representative of three independent experiments (mean ± s.d. of $n = 3$ co-cultures).*p<0.05, **p<0.01, ***p<0.001 (paired two-tailed Student's t-test). Red indicates anti-FLAG *versus* isotype; blue indicates anti-HA *versu*s isotype. (G) Representative flow plots for the data from (E,F).

Supplementary Figure 2

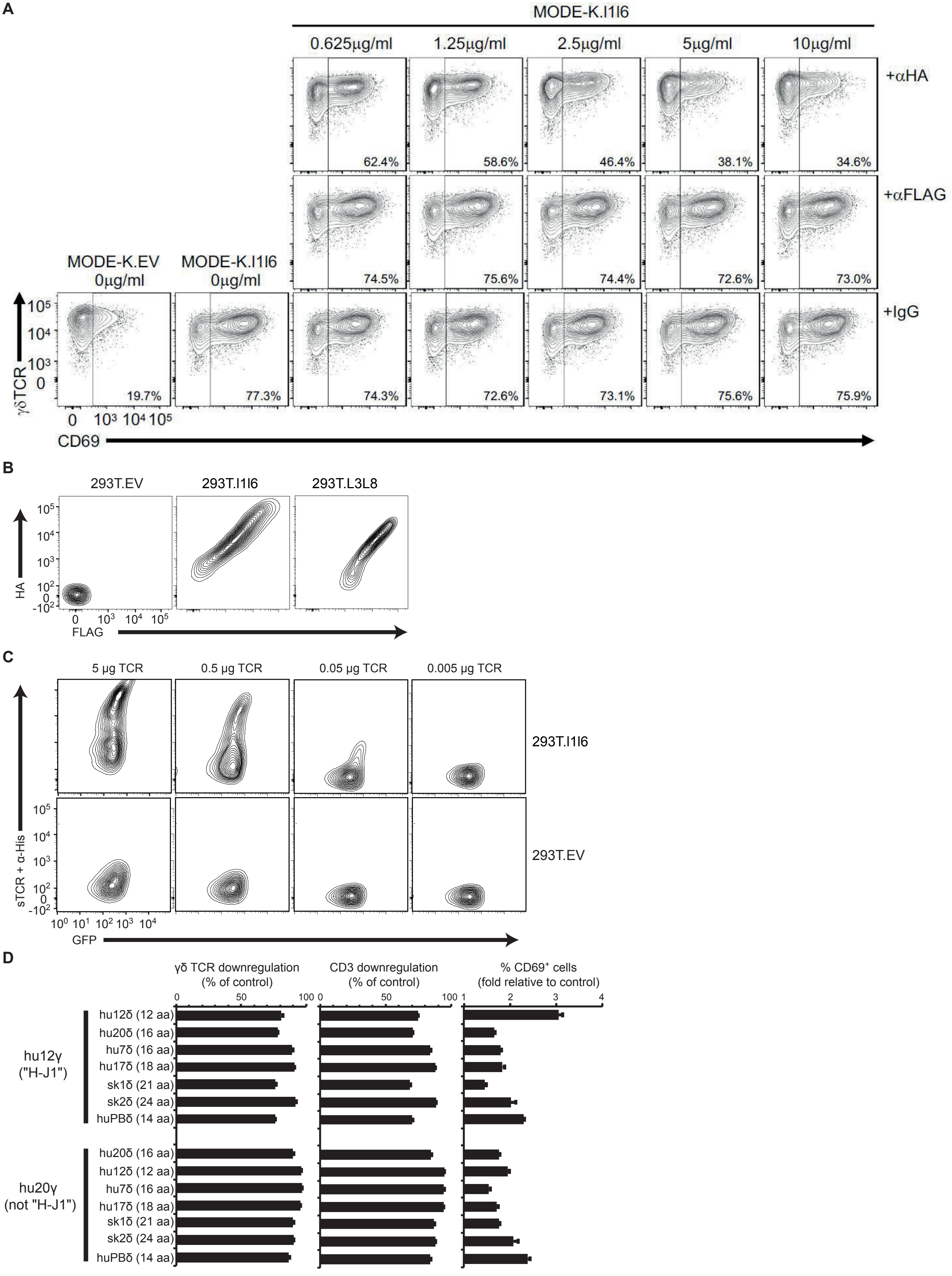

Supplementary Figure 2 - related to main text Figure 2. (A) Flow cytometry analysis of TCR downregulation and CD69 upregulation by Jurkat 76 cells transduced with mo6 Vγ7Vδ2-2 TCR and co-cultured with MODE-K.FLAG-l1.HA-l6 cells in the presence of the indicated concentrations of antibodies (x-axis). Data are representative of three independent experiments. (B) Flow cytometry analysis of 293T cells transduced to express FLAG-l1.HA-l6 or FLAG-L3.HA-L8, stained with anti-FLAG and anti-HA antibody. (C) Flow cytometry of 293T.l1l6 or 293T.EV cells stained with the indicated concentrations of soluble mouse Vγ7Vδ7 TCR and anti-His mAb complexes. (D)Flow cytometry analysis of CD3/TCR downregulation and CD69 upregulation by JRT3 cells expressing hu12γ (contains the H-J1 motif) or hu20γ (does not contain the H-J1 motif) paired with Vδ1 chains of varying CDR3 lengths and sequences (Melandri et al., 2018) co-cultured with 293T.L3L8 for 3h. Results were normalised to those obtained by co-culture with 293T.EV cells. Data are representative of three independent experiments (mean ± s.d. of n = 3 co-cultures).

Supplementary Figure 3

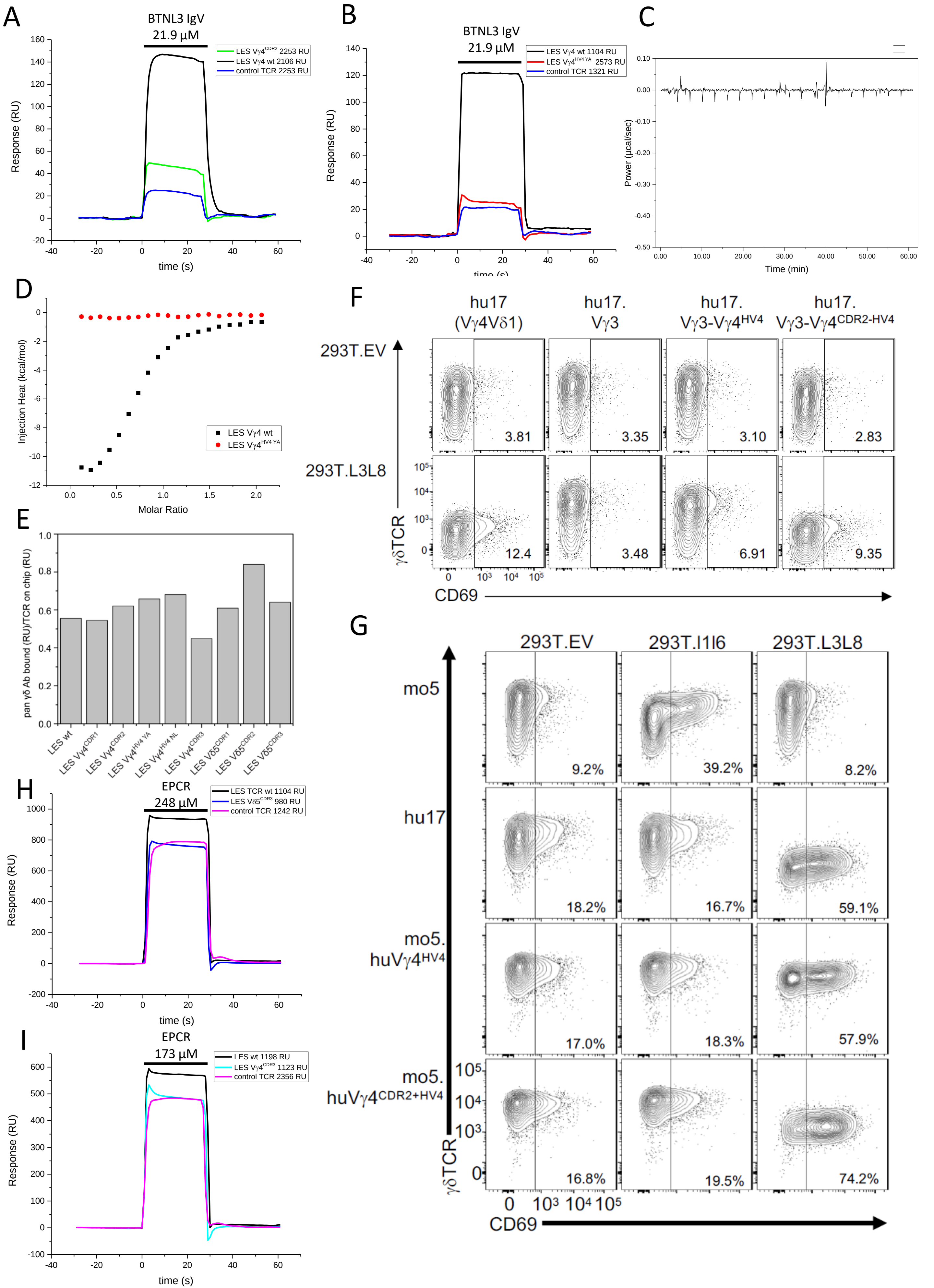

Supplementary Figure 3 - related to main text Figure 3. (A-B) Representative SPR analysis of BTNL3 IgV (21.9μM) injected (horizontal bar) over (A) LES Vγ4 wt, LES Vγ4^{CDR2} mutant, and control TCR, or (B) LES Vγ4 wt, LES Vγ4$^{HV4\ YA}$ mutant, and control TCR. (C,D) ITC analysis indicates negligible binding between BTNL3 and the LES Vγ4^{YA} TCR mutant. (E) Binding of anti-γδ TCR mAb 11F2 (RU) relative to amount of wt or mutant TCR on the chip surface (RU). (F) Representative flow plots for the data shown in Figure 3C. (G) Representative flow plots for the data shown in Figure 3D,E. (H-I) Representative SPR analysis of EPCR (248 μM or 173 μM) injected (horizontal bar) over (H) wt LES TCR, LES Vδ5^{CDR3} mutant, and control TCR, or (I) wt LES TCR, LES Vγ4^{CDR3} mutant, and control TCR.

Supplementary Figure 4

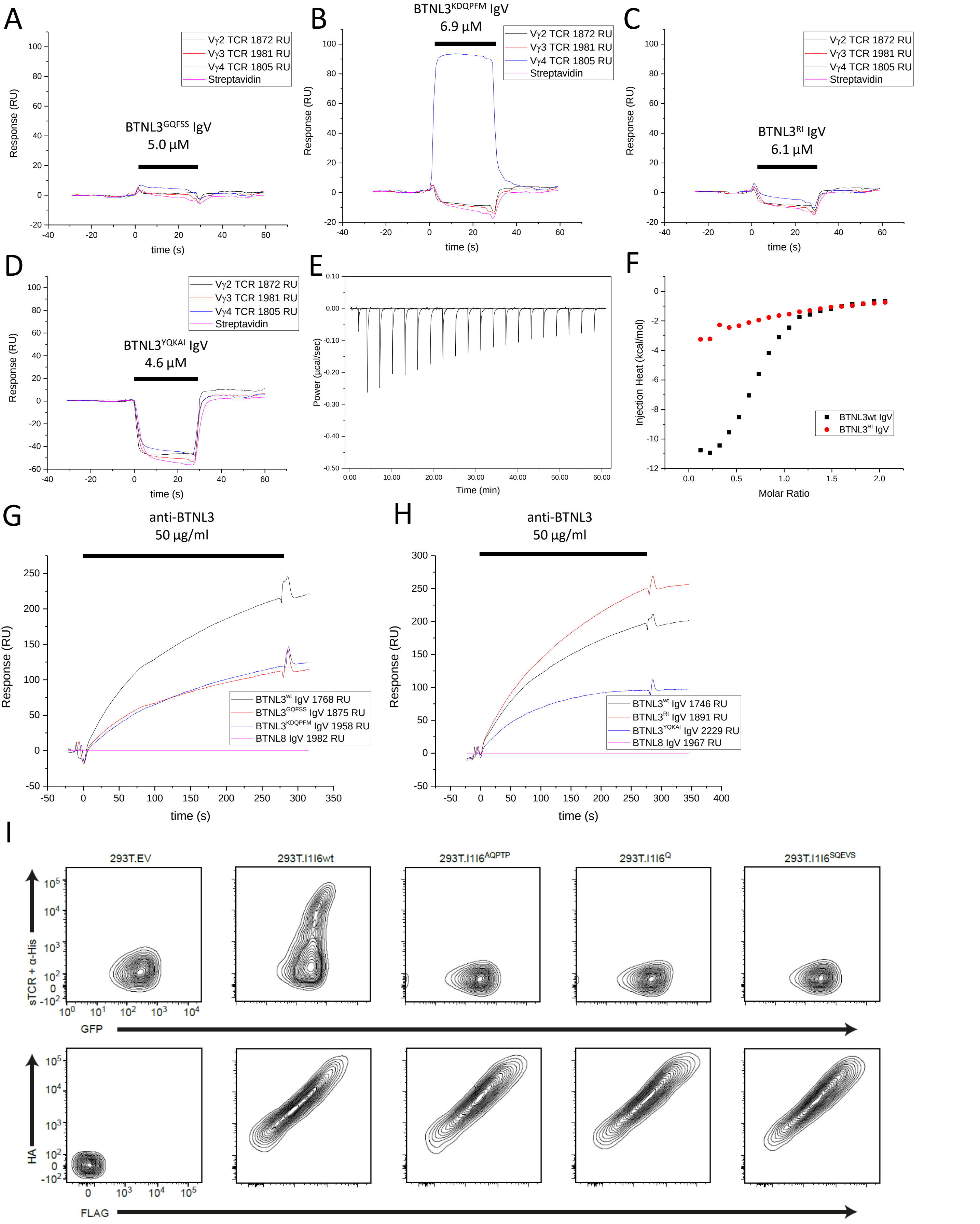

J

V-shaped dimer interface contacts based on BTN3A1 ectodomain crystal lattice (PDB entry 4F80)

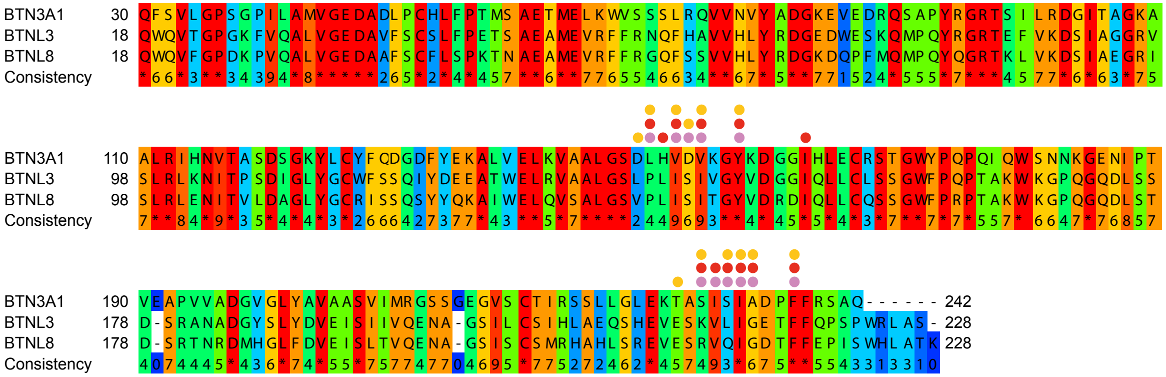

K

Head-to-tail dimer interface contacts based on BTN3A1 ectodomain crystal lattice (PDB entry 4F80)

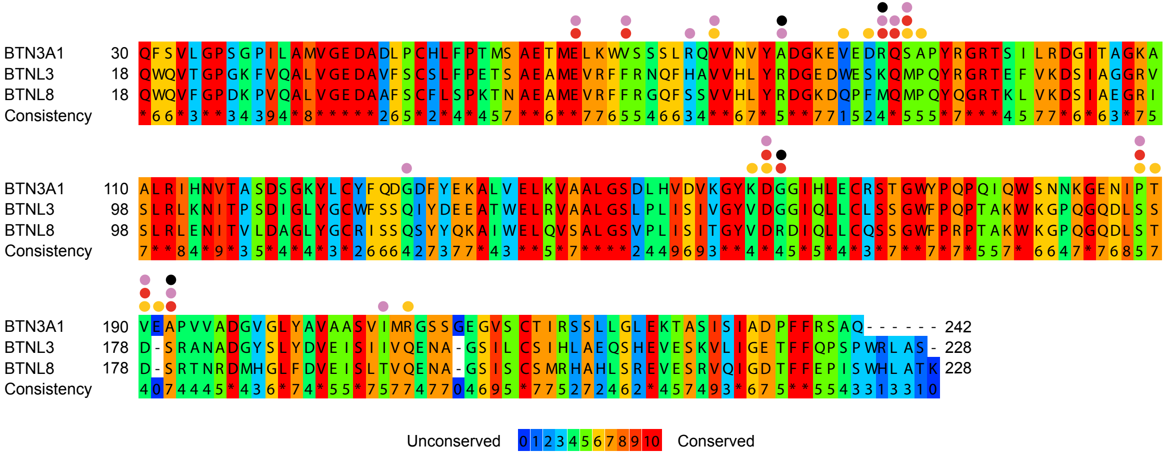

Supplementary Figure 4 - related to main text Figure 4. Vγ4+ TCR interaction with BTNL3 mutants (A-D) Negligible binding of BTNL3^{GQFSS} (A), BTNL3^{RI} (C) and BTNL3^{YQKAI} (D) mutants injected (horizontal bar) in HBS-EP over immobilised Vγ4 or control TCRs, whereas BTNL3^{KDQPFM} binds with an affinity of 11.2μM. Negative signals reflect residual amounts of Tris buffer in which purified protein was stored. (E-F) ITC analysis showing injection of BTNL3^{RI} mutant into solution containing wt LES Vγ4 TCR (K_d~50μM). (G,H) Binding of anti-BTNL3 antibody to wt or mutant BTNL3 IgV proteins immobilised via His tag to a NTA Sensor Chip. (I) Vγ7+ TCR/anti-His staining of 293T target cells expressing Btnl1 and either wt or mutant Btnl6. (J-K). BTNL3.8 ectodomain is likely to adopt a V-shaped dimer configuration observed in the BTN3A1 crystal lattice. (J) Alignment of IgV-IgC sequences from human BTN3A1, BTNL3 and BTNL8. Sequences were retrieved from Uniprot (accession numbers 000481 (BTN3A1), Q6UXE8 (BTNL3) and Q63UX41 (BTNL8). Alignment was performed using the PRALINE multiple sequence alignment toolkit (Bawono and Heringa, 2014) with the colour scheme showing the degree of amino acid conservation. Residues that contribute to stabilising the V-shaped and head to tail dimer interface are shown for BTN3A1 (yellow circles), BTNL3 (pink circles) and BTNL8 (red circles). Residues that stabilise the V-shaped dimer are relatively highly conserved between BTN3A1 and BTNL3 and BTNL8, whereas side chains that contribute to the head-to-tail interface are less well conserved. (K) Residues that may prevent the formation of the BTNL3/BTNL8 head-to-tail heterodimer due to steric and/or electrostatic incompatibility are highlighted (black circles).

Supplementary Figure 5

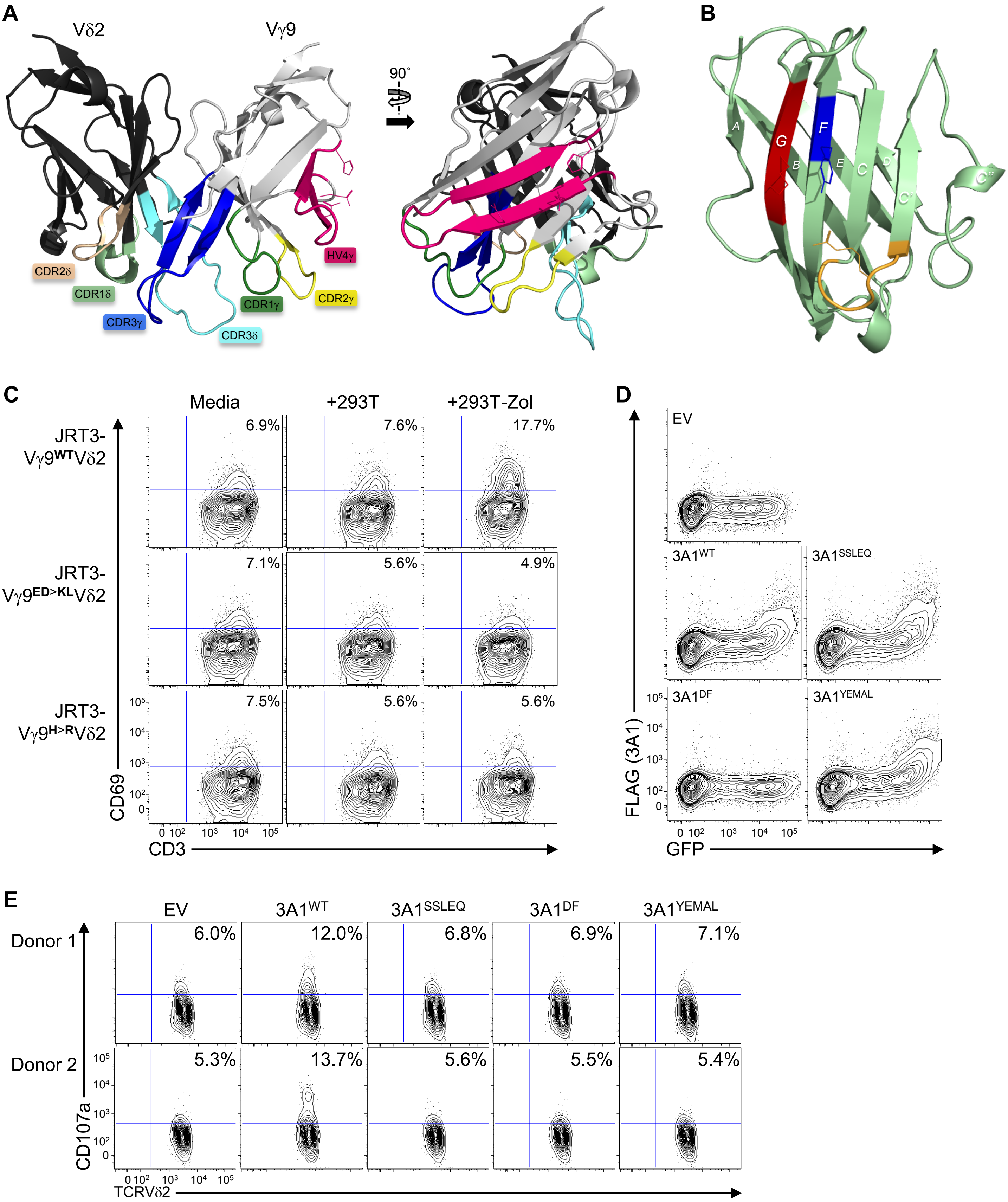

F

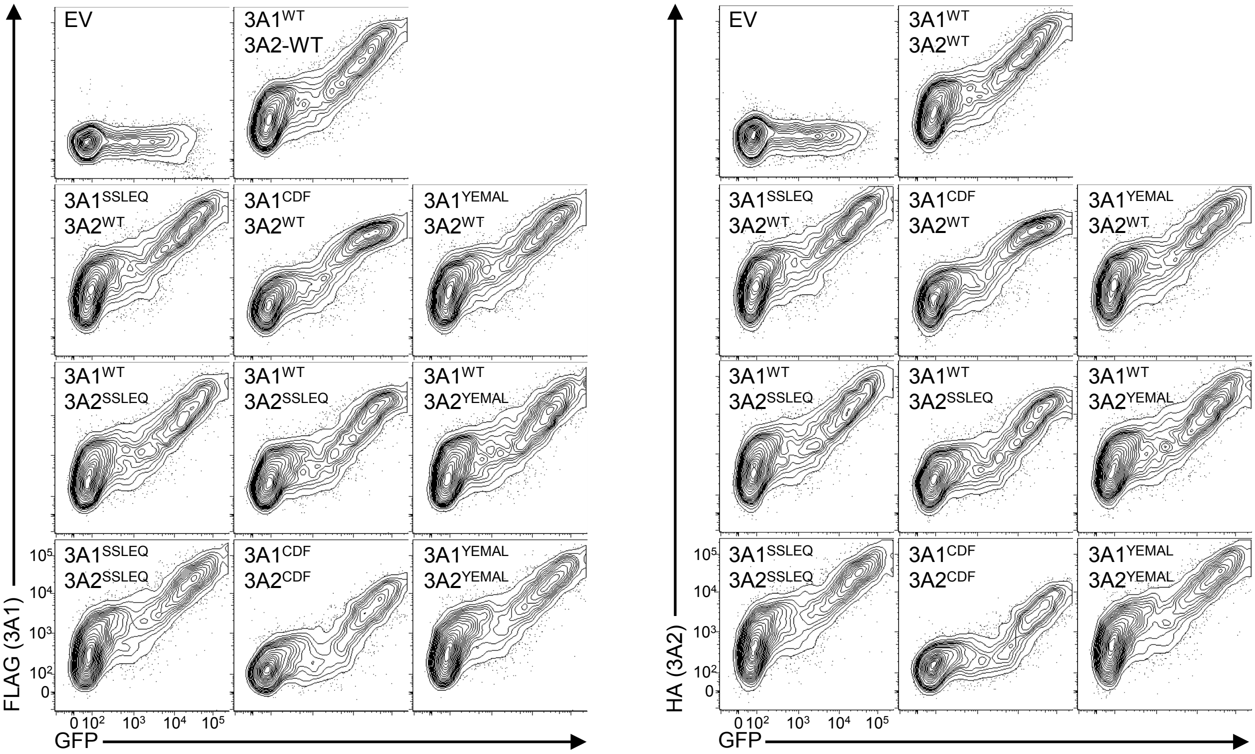
EV
3A1WT
3A2-WT
3A1SSLEQ
3A2WT
3A1CDF
3A2WT
3A1YEMAL
3A2WT
3A1WT
3A2SSLEQ
3A1WT
3A2SSLEQ
3A1WT
3A2YEMAL
3A1SSLEQ
3A2SSLEQ
3A1CDF
3A2CDF
3A1YEMAL
3A2YEMAL
FLAG (3A1)
HA (3A2)
GFP
0 10² 10³ 10⁴ 10⁵

G

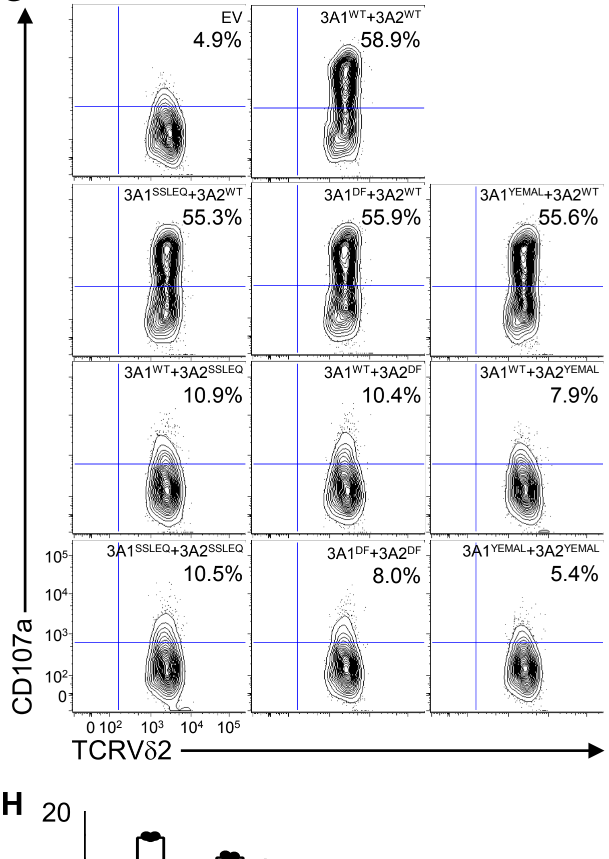
EV 4.9%
3A1WT+3A2WT 58.9%
3A1SSLEQ+3A2WT 55.3%
3A1DF+3A2WT 55.9%
3A1YEMAL+3A2WT 55.6%
3A1WT+3A2SSLEQ 10.9%
3A1WT+3A2DF 10.4%
3A1WT+3A2YEMAL 7.9%
3A1SSLEQ+3A2SSLEQ 10.5%
3A1DF+3A2DF 8.0%
3A1YEMAL+3A2YEMAL 5.4%
CD107a
TCRVδ2

I

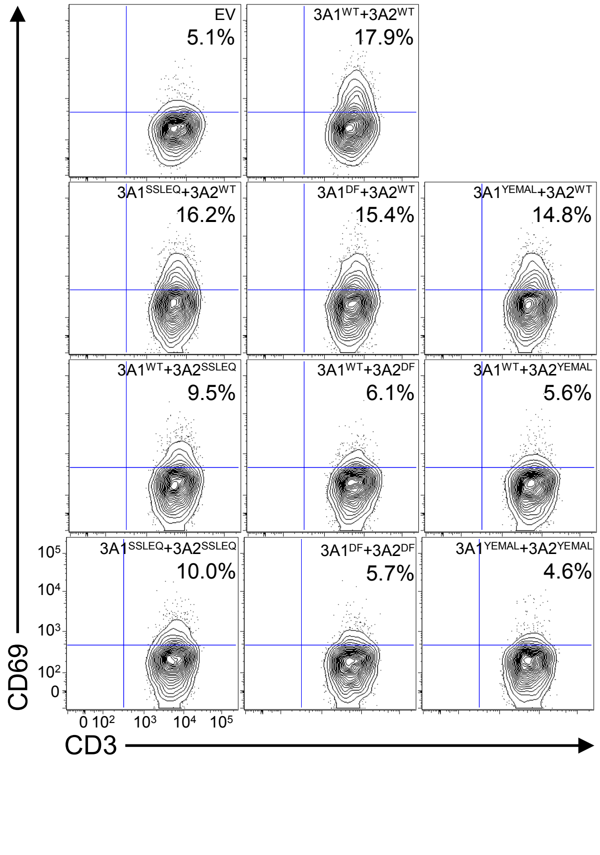
EV 5.1%
3A1WT+3A2WT 17.9%
3A1SSLEQ+3A2WT 16.2%
3A1DF+3A2WT 15.4%
3A1YEMAL+3A2WT 14.8%
3A1WT+3A2SSLEQ 9.5%
3A1WT+3A2DF 6.1%
3A1WT+3A2YEMAL 5.6%
3A1SSLEQ+3A2SSLEQ 10.0%
3A1DF+3A2DF 5.7%
3A1YEMAL+3A2YEMAL 4.6%
CD69
CD3

H

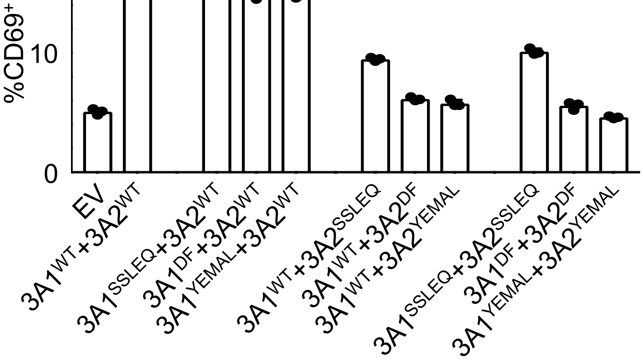
%CD69+
20
10
0
EV
3A1WT+3A2WT
3A1SSLEQ+3A2WT
3A1DF+3A2WT
3A1YEMAL+3A2WT
3A1WT+3A2SSLEQ
3A1WT+3A2DF
3A1WT+3A2YEMAL
3A1SSLEQ+3A2SSLEQ
3A1DF+3A2DF
3A1YEMAL+3A2YEMAL

Supplementary Figure 5 - related to main text Figure 5. (A) Cartoon representation of the G115 Vγ9Vδ2 TCR V-domain structure (from PDB accession code 1HXM), with all CDRs and HV4γ highlighted. Side chains are displayed for the amino acids of interest (see Figure 4B). (B) Cartoon representation of the BTN3A1 IgV domain structure (from PDB accession code 4F80). The SSLQE, YF and YEKAL motifs are highlighted in orange, blue and red, respectively. Side chains are displayed for the amino acids of interest (see Figure 6C). (C) Representative flow cytometry analysis of CD69 upregulation by JRT3 cells expressing the indicated Vγ9Vδ2 TCR constructs following incubation with media only, or 293T cells with or without pre-treatment with Zoledronate (Zol, 10 μM). Related to Figure 6E. (D) Representative flow cytometry analysis of the expression of the indicated FLAG-tagged BTN3A1 constructs (EV, empty vector control) 48 h post-transfection in 293T cells. (E) Representative flow-cytometry analysis of CD107a upregulation by polyclonal Vγ9Vδ2 T cell lines derived from PBMCs from two donors following co-culture with CRA123 cells transfected with the indicated BTN3A1 constructs or empty vector control (EV) and pre-treated with 10 μM Zol. (F) Representative flow-cytometry analysis of the expression of the indicated FLAG-tagged BTN3A1 and HA-tagged BTN3A2 constructs or empty vector control (EV) 48 h post-co-transfection in 293T cells. (G) Representative flow-cytometry analysis of CD107a upregulation by a polyclonal Vγ9Vδ2 T cell line following co-culture with CRA123 cells co-transfected with the indicated BTN3A1 and BTN3A2 constructs or empty vector control (EV) and pre-treated with 10 μM Zol. Related to Figure 5G. (H,I) Flow-cytometry analysis of CD69 upregulation by JRT3 cells expressing a wild-type Vγ9Vδ2 TCR following co-culture with CRA123 cells co-transfected with the indicated BTN3A1 and BTN3A2 constructs or empty vector control (EV) and pre-treated with 10 μM Zol. Data in (H) are representative of two independent experiments (mean ± s.d. of n = 3 co-cultures). Corresponding representative flow-cytometry plots are shown in (I).
